# Utility of miRNA biomarkers in patients with acute myocardial infarction (UNIAMI)—results from a prospective pilot study

**DOI:** 10.3389/fcvm.2026.1730999

**Published:** 2026-06-11

**Authors:** Chakradhara Rao S. Uppugunduri, Koustav Sarkar, Oishi Mukherjee, Kaviya Manoharan, Iyshwarya B. K, Melvin George

**Affiliations:** 1 Department of Medical Oncology, Jawaharlal Institute of Postgraduate Medical Education and Research, Puducherry, India; 2 Department of Pediatrics, Gynecology and Obstetrics, CANSEARCH research platform of Pediatric Oncology and Hematology of University of Geneva, Geneva, Switzerland; 3 Department of Biotechnology, School of Bioengineering, College of Engineering and Technology, SRM Institute of Science and Technology, Chennai, India; 4 Centre for Clinical Pharmacology, SRM Medical College Hospital & Research Centre, Kattankulathur, India

**Keywords:** acute myocardial infarction, biomarkers, circulating microRNAs, coronary artery disease, PCA clustering, ROC analysis, South Asian population

## Abstract

**Background:**

Acute myocardial infarction (AMI) is a leading cause of death worldwide, occurring earlier and more severely in South Asian populations. Circulating microRNAs (miRNAs) are stable, non-invasive biomarkers with emerging diagnostic and prognostic potential, but population-specific data from South India remain limited.

**Methods:**

In this prospective cohort, 216 participants were enrolled: 45 AMI patients, 71 stable coronary artery disease (CAD) patients, and 100 healthy controls. Plasma samples were collected within 24 h of symptom onset in AMI cases. Expression of prioritized circulating miRNAs (*n* = 20) was quantified using qRT-PCR SYBR green chemistry using specific primers. Statistical analyses included hierarchical and k-means clustering, principal component analysis (PCA), pathway enrichment, and receiver operating characteristic (ROC) curve analyses.

**Results and conclusion:**

MI patients showed significant upregulation of let-7b, let-7c-5p, miR-24-1, miR-342-3p, and miR-362-3p, and downregulation of miR-4485-3p, miR-494-3p, miR-939, and miR-4505 (*p* < 0.05). Cluster and PCA analyses revealed distinct segregation of AMI cases, while CAD exhibited intermediate molecular profiles. Pathway enrichment implicated ECM–receptor interaction, platelet activation, and PI3K/AKT signaling. ROC analysis demonstrated excellent discriminatory performance, with miR-4485-3p achieving an AUC of 1.000. There were no significant differences in the expression pattern of miR-3195, miR-6780b-5p between CAD and AMI patients. These findings highlight circulating miRNAs as promising biomarkers for AMI, reflecting key molecular processes of inflammation, apoptosis, and endothelial dysfunction.

## 1 Introduction

Cardiovascular disorders affect the heart and/or blood vessels and remain the leading cause of death worldwide, despite significant advancements in diagnosis and treatment (1). Understanding the underlying cellular and molecular mechanisms may contribute to the prevention of cardiovascular diseases. Acute myocardial infarction (AMI) continues to be one of the most serious forms of coronary artery disease (CAD) around the world. Recent demographic figures show that CAD has a major impact on public health around the world. Approximately 127.9 million Americans (48.6% of those aged 20 and older) suffer from cardiovascular disease (CVD), which includes heart failure, stroke, and CAD, according to the 2024 heart disease and Stroke Statistics. CAD is the most common form of heart disease in the US, affecting approximately 28.6 million adults (2). It continues to be one of the leading causes of mortality and morbidity worldwide, as well as in India. In the South Asian population, deaths due to AMI occurred five to ten years earlier than in the Western population, and AMI occurred among men who were 5.6 years younger than women (3). ST-Elevation Myocardial Infarction (STEMI) is a clinical syndrome defined by characteristic symptoms of myocardial ischemia in association with persistent electrocardiographic (ECG) ST-segment elevation and subsequent release of biomarkers of myocardial necrosis (4).

MicroRNAs are small, non-coding RNA molecules that regulate gene expression at the post-transcriptional level by binding to complementary sequences on target messenger RNAs (mRNAs). They are involved in various cellular processes, including apoptosis, proliferation, and differentiation, making them critical players in cardiovascular health and disease (5). The stability of circulating miRNAs in body fluids, combined with their tissue-specific expression patterns, positions them as ideal biomarkers for various diseases, including cardiovascular conditions (6, 7).

Several studies have shown that patients with AMI exhibit altered miRNA signatures, indicating their potential for early detection, risk assessment, and monitoring of cardiovascular diseases. However, there is a lack of population-specific data regarding circulating miRNA profiles, especially among South Indian individuals with AMI. Previous prospective cohort studies have further demonstrated the diagnostic and prognostic utility of circulating miRNAs, such as miR-1 in chest pain patients and miR-208b/miR-34a in predicting post-infarction remodelling, underscoring their clinical relevance (8). However, there is a lack of population -specific data regarding circulating miRNA profiles, especially among South Indian individuals with AMI. This study aims to profile prioritized circulating miRNAs in patients with AMI, those with stable CAD, and healthy controls. The goal is to explore their diagnostic potential and enhance our understanding of the molecular mechanisms involved in myocardial infarction within this population.

## 2 Materials and methods

### 2.1 Study population

A total of 216 participants were enrolled in this prospective cohort study, including 45 patients diagnosed with AMI, 71 patients with stable CAD with no previous history of acute coronary events, and 100 healthy controls. Participants were screened and recruited from the Intensive Care Unit (ICU) of the Department of Cardiology at SRM Medical College Hospital and Research Centre, Kattankulathur, Tamil Nadu, India, between September 2023 and March 2024. Eligibility criteria for cases included a diagnosis of AMI according to the American Heart Association/American College of Cardiology (AHA/ACC) 2014 guidelines, age ≥18 years, South Indian ancestry (residing in South India for at least three generations), and willingness to provide written informed consent. Healthy controls were individuals with no history of cardiovascular disease, cancer, chronic kidney disease, or active/autoimmune disorders. Patients with terminal illnesses or those presenting more than 72 h after symptom onset were excluded. All follow-up data were collected by a trained clinical research associate. The study protocol was approved by the Institutional Ethics Committee (IEC No: 8676) of SRM Medical College Hospital and Research Centre, and written informed consent was obtained from all participants before enrollment.

### 2.2 Sample collection and processing

All participants were screened according to predefined inclusion and exclusion criteria. Healthy controls, matched by age, were recruited from among hospital visitors and were confirmed to have no clinical history of cardiovascular disease or other chronic illnesses. Upon obtaining written informed consent, 4 mL of peripheral venous blood was collected from the antecubital vein using ethylenediaminetetraacetic acid (EDTA) tubes. For patients with AMI, blood samples were drawn within 24 h of symptom onset, and the exact time of collection was recorded. The collected blood was centrifuged at 2000 g for 10 min at 4 °C to separate plasma. The resulting plasma was divided into three 500 μL aliquots and stored at −80°C until further analysis of selected microRNA (miRNA) candidates.

### 2.3 Isolation of RNA from human plasma

Total RNA was isolated from 50 to 250 μL of plasma using the Trizol-based method (9). Briefly, 750 μL of Trizol reagent was added to each plasma sample, mixed by gentle inversion (5–8 times), and incubated at room temperature for 15 min. Then, 200 μL of chloroform was added, followed by vigorous inversion for 15 s and a further incubation at room temperature for 5 min. Phase separation was achieved by centrifugation at 12,000 × g for 15 min at 4°C. The upper aqueous phase was carefully collected and transferred to a new RNase-free tube. RNA was precipitated using 500 μL of isopropanol, incubated for 10 min at room temperature, and centrifuged at 12,000 × g for 10 min at 4°C. The resulting RNA pellet was washed with 75% ethanol, air-dried at room temperature for approximately 30 min, and finally resuspended in 20 μL of RNase-free water (Qiagen, Hilden, Germany). RNA concentration and purity were assessed using a NanoDrop ND-1,000 UV-Vis spectrophotometer by measuring absorbance at A260/280 and A260/230 nm.

### 2.4 Conversion of RNA into cDNA

Complementary DNA (cDNA) was synthesized using the High-Capacity cDNA Reverse Transcription Kit (Applied Biosystems) according to the manufacturer's instructions. The reverse transcription protocol included: 5 min at 25°C for primer annealing, 120 min at 37°C for reverse transcription, and 1 min at 95°C for enzyme inactivation. The resulting cDNA was stored at −20°C until further use.

### 2.5 Analysis of miRNA gene expression by qRT-PCR

Quantitative real-time PCR reactions were set up using the SYBR Green master mix. The temperature program for the real-time PCR analysis was as follows: 50°C for 2 min (1 cycle), 95°C for 10 min (1 cycle), 95°C for 15 s -> 60°C for 30 s -> 72°C for 30 s (40 cycles), and finally 72°C for 10 min (1 cycle). Dissociation/melt curve analysis was included in the amplification protocol. Normalization of the results was performed using the expression levels of miU6. The miRNA gene-specific primers used are provided in Table 1. Efficiency testing of the internal control gene and test primers set was performed using pooled total RNA from 10 anonymized healthy volunteers in three different dilutions and the efficiency of all the primers tested was in the range of 98%–106%.

**Table 1:** Designed primer sequences for selected miRNAs.

S.No	miRNAs	Seq 5’ to 3’	Length bp	Tm
1.	miR-4505 (F)	AGGCTGGGCTGGGACG	16	59.43
	miR-4505 (R)	GAACATGTCTGCGTATCTC	19	54.57
2.	Let-7c-5p (F)	TGAGGTAGTAGGTTGTATGGTT	22	56.53
	Let-7c-5p (R)	CAGTGCGTGTCGTGGAGT	18	58.24
3.	miR-6875-5p (F)	ACTGCGTGAGGGACCCA	17	57.60
	miR-6875-5p (R)	ACGCTCAGTTAATGCTAATCGTGATA	26	60.07
4.	miR-939 (F)	GGGAGCTGAGGCTCTG	16	56.86
	miR-939 (R)	GAACATGTCTGCGTATCTC	19	54.51
5.	Let-7b (F)	GAGGTAGTAGGTTGTGTG	18	53.69
	Let-7b (R)	GAACATGTCTGCGTATCTC	19	54.51
6.	miR-4485-3p (F)	TAACGGTCGCGGTACCCTAA	20	59.35
	miR-4485-3p (R)	AGACTGGATGAGACTGTGACTTG	23	60.65
7.	miR-3195 (F)	CGCGCCGGGCCCGGG	15	66.97
	miR-3195 (R)	GAACATGTCTGCGTATCTC	19	54.51
8.	miR-7641-1 (F)	TTGATCTCGGAAGCTAAGC	19	54.51
	miR-7641-1 (R)	GAACATGTCTGCGTATCTC	19	54.51
9.	miR-494-3p (F)	AACGAGACGACGACAGAC	18	55.97
	miR-494-3p (R)	TGAAACATACACGGGAAACCTC	22	58.39
10.	miR-33a (F)	GTGCATTGTAGTTGCATTG	19	52.35
	miR-33a (R)	GAACATGTCTGCGTATCTC	19	54.51
11.	miR-24-1 (F)	GCCTACTGAGCTGATATC	18	53.69
	miR-24-1 (R)	GAACATGTCTGCGTATCTC	19	54.51
12.	miR-548a-5p (F)	GGGAAAAGTAATTGCGAG	18	51.41
	miR-548a-5p (R)	CAGTGCGTGTCGTGGA	16	54.30
13.	miR-101 (F)	CAGTTATCACAGTGCTGA	18	51.41
	miR-101 (R)	GAACATGTCTGCGTATCTC	19	54.51
14.	miR-142-3p (F)	GGGGGTGTAGTGTTTCCTA	19	56.67
	miR-142-3p (R)	CAGTGCGTGTCGTGGA	16	54.30
15.	miR-362-3p (F)	AACACACCTATTCAAGGATTCA	22	54.66
	miR-362-3p (R)	ACGTGACACGTTCGGAGAATT	21	57.87
16.	miR-29b (F)	GCTGGTTTCATATGGTGG	18	53.69
	miR-29b (R)	GAACATGTCTGCGTATCTC	19	54.51
17.	miR-342-3p (F)	TCCTCGCTCTCACACACAGAAATC	22	60.25
	miR-342-3p (R)	TATGGTTGTTCACGACTCCTTCAC	24	61.01
18.	miR-32-5p (F)	GCGGCGTATTGCACATTACT	20	57.30
	miR-32-5p (R)	TCGTATCCAGTGCAGGGTC	19	58.82
19.	miR-6780b-5p (F)	CAGCCTGGGGAAGGCTTG	18	60.52
	miR-6780b-5p (R)	AAAGGAGACAAGGGAGAGGC	20	59.35
20.	miR U6 (F)	ATTGGAACGATACAGAGAAGATT	23	55.30
	miR U6 (R)	GGAACGCTTCACGAATTTG	19	54.51

### 2.6 Statistical analysis

Participants were categorized into two primary groups: acute myocardial infarction (AMI) patients and stable CAD controls. Health controls were included solely to explore relative differences in the measured biomarkers among healthy individuals to understand their predictive nature for any CAD or AMI event. The distribution of continuous variables was assessed visually using histograms. Continuous variables were summarized as mea*n* ± standard deviation (SD) or median with interquartile range (IQR), depending on data normality. Categorical variables were presented as frequencies and percentages. Differences between groups for categorical data were analyzed using the chi-square test. For continuous variables, the Mann–Whitney *U*-test or the Independent Samples *t*-test was applied, depending on the normality of distribution. To explore expression patterns of circulating miRNAs, unsupervised hierarchical clustering and k-means clustering (*k* = 3) were performed on normalized *Δ*Ct values. Principal Component Analysis (PCA) was conducted to reduce dimensionality and to visualize group-level segregation; the percentage of variance explained by each principal component was recorded. The discriminatory power of individual miRNAs was evaluated using Receiver Operating Characteristic (ROC) curve analysis, with calculation of Area Under the Curve (AUC), 95% confidence intervals (CI), and *p*-values. AUC values >0.8 were considered to indicate strong discriminatory ability. All statistical analyses were performed using R studio 2025.0 build 496 with R version 4.4.2. Two-sided adjusted *p*-values < 0.05 were considered statistically significant, with correction applied for multiple comparisons to minimize false positives using Bonferroni correction test. Some clinical variables had missing data, primarily due to incomplete documentation during initial admission or follow-up. No imputation was performed; all analyses were conducted using available (complete case) data.

## 3 Results

### 3.1 Baseline clinical characteristics and biochemical parameters of the study population

A total of 116 participants were analyzed, comprising 45 AMI cases and 71 CAD controls. Baseline clinical parameters are presented in Table 2. Significant differences were seen in height and BMI among AMI vs. CAD controls (*p* < 0.001), whereas no significant differences were seen in age (*p* = 0.96), weight (*p* = 0.41) between the two groups. Among the biochemical parameters, FBS was significantly higher in the AMI (189.57 ± 73.40 mg/dL) than CAD (151.91 ± 80.41 mg/dL), with *p* = 0.01. Blood urea nitrogen (BUN) was also significantly elevated in AMI (10.33 ± 2.08 mg/dL vs. 0.83 ± 3.28 mg/dL) with a *p*-value of <0.0001. Creatine phosphokinase-MB (CPK-MB) levels were markedly elevated in AMI patients (147.10 ± 181.74 IU/L) compared to CAD controls (38.83 ± 36.55 IU/L; *p* < 0.0001), consistent with acute myocardial injury. Diastolic blood pressure (DBP) was also higher in AMI cases (82.07 ± 14.97 mmHg vs. 76.75 ± 13.16 mmHg; *p* = 0.04), while pulse rate (PR) was lower (72.21 ± 26.42 bpm vs. 83.62 ± 13.33 bpm; *p* = 0.002). Other biochemical parameters did not seem to be associated with the two groups.

**Table 2:** Comparison of the baseline characteristics of AMI cases and CAD controls.

Baseline	AMI (*N* = 45)	CAD (*N* = 71)	*p*-Value
Age (yrs)	56.93 ± 11.73	57.01 ± 9.77	0.96
Height (cms)	175.50 ± 23.33	160.50 ± 9.14	<0.0001
Weight (kgs)	62.06 ± 7.41	63.53 ± 10.61	0.41
BMI (kg/m^2^)	21.20 ± 1.92	24.85 ± 5.39	<0.0001
Blood Glucose	164.95 ± 62.12	153.54 ± 82.66	0.42
Urea	26.65 ± 11.54	29.08 ± 14.05	0.33
BUN (mg/dl)	10.33 ± 2.08	0.83 ± 3.28	<0.0001
Creatinine (mg/dl)	0.94 ± 0.35	0.93 ± 0.34	0.87
FBS (mg/dl)	189.57 ± 73.40	151.91 ± 80.41	0.01
HbA1c (%)	7.86 ± 2.20	7.95 ± 2.26	0.83
Total Cholesterol (mg/dl)	179.58 ± 42.77	174.14 ± 42.90	0.50
HDL (mg/dl)	51.67 ± 38.13	43.59 ± 24.75	0.16
LDL (mg/dl)	120.64 ± 43.50	121.04 ± 31.59	0.95
VLDL (mg/dl)	27.05 ± 12.56	28.50 ± 20.50	0.67
TGL (mg/dl)	138.67 ± 50.25	128.61 ± 72.48	0.41
CPK (iu/l)	360.40 ± 276.29	444.17 ± 510.83	0.31
CPKMB (iu/l)	147.10 ± 181.74	38.83 ± 36.55	<0.0001
Hb (g/dl)	12.31 ± 3.20	12.18 ± 2.10	0.79
Total Cells (ml)	9,935.56 ± 2,694.16	–	–
PCV (%)	42.46 ± 16.12	39.00 ± 8.55	0.13
SBP (mmHg)	127.93 ± 24.84	121.38 ± 13.35	0.06
DBP (mmHg)	82.07 ± 14.97	76.75 ± 13.16	0.04
PR ((bpm)	72.21 ± 26.42	83.62 ± 13.33	0.002

### 3.2 Comparison of lifestyle and dietary patterns among AMI vs CAD

The distribution of demographic and lifestyle factors is summarized in Table 3. There were no significant differences between AMI and CAD groups in terms of gender (*p* = 0.39), diabetes (*p* = 0.22), hypertension (*p* = 0.44), smoking (*p* = 0.13), alcohol use (*p* = 0.31), or family history of cardiovascular disease (*p* = 0.70). However, several dietary habits differed significantly. Chicken liver consumption was more frequent among AMI patients (*p* < 0.0001), followed by red meat (*p* = 0.001) and cauliflower (*p* = 0.0002), suggesting potential links between specific dietary patterns and AMI risk. Other dietary items, including milk, eggs, seafood, chicken, greens, beetroot, and soybeans, did not differ significantly between the groups (*p* > 0.05). These findings highlight dietary variation as a potentially important environmental risk factor.

**Table 3:** Comparison of the demographic and dietary patterns of AMI cases and CAD controls.

Demographics	AMI (*N* = 45)	CAD (*N* = 71)	*p*-value
*Gender*			
Male	32 (71.1)	45 (63.4)	0.39
Female	13 (28.9)	26 (36.6)	
*Diabetes*			
Yes	19 (42.2)	25 (35.2)	0.22
No	21 (46.7)	45 (63.4)	
*Hypertension*			
Yes	21 (46.7)	29 (40.8)	0.44
No	22 (48.9)	41 (57.7)	
*Smoking*			
Yes	13 (28.9)	14 (19.7)	0.13
No	27 (60.0)	57 (80.3)	
*Hypercholesterolemia*			
Yes	7 (15.6)	12 (16.9)	0.89
No	31 (68.9)	57 (80.3)	
*Family History*			
Yes	9 (20.0)	18 (25.4)	0.70
No	31 (68.9)	52 (73.2)	
*Alcohol*			
Yes	15 (33.3)	20 (28.2)	0.31
No	25 (55.6)	51 (71.8)	
*Dietary Pattern*			
Daily	5 (11.1)	7 (9.9)	
Weekly once	40 (88.9)	62 (87.3)	
Monthly once	0 (0)	0 (0)	0.87
Occasionaly	0 (0)	0 (0)	
Never	0 (0)	0 (0)	
*Milk*			
Daily	41 (91.1)	63 (88.7)	
Weekly once	1 (2.2)	2 (2.8)	
Monthly once	0 (0)	0 (0)	0.93
Occasionaly	2 (4.4)	4 (4.2)	
Never	1 (2.2)	3 (4.2)	
*Egg*			
Daily	8 (17.8)	4 (5.6)	
Weekly once	25 (55.6)	43 (60.6)	
Monthly once	2 (4.4)	2 (2.8)	0.23
Occasionaly	5 (11.1)	14 (19.7)	
Never	5 (11.1)	7 (9.9)	
*Sea food*			
Daily	2 (4.4)	1 (1.4)	
Weekly once	28 (62.2)	38 (53.5)	
Monthly once	4 (8.9)	11 (15.5)	0.63
Occasionaly	5 (11.1)	10 (14.1)	
Never	6 (13.3)	11 (15.5)	
*Chicken*			
Daily	2 (4.4)	0 (0)	
Weekly once	23 (51.1)	44 (62.0)	
Monthly once	4 (8.9)	7 (9.9)	0.33
Occasionaly	5 (11.1)	8 (11.3)	
Never	11 (24.4)	12 (16.9)	
*Liver*			
Daily	8 (17.8)	1 (1.4)	
Weekly once	25 (55.6)	14 (19.7)	
Monthly once	2 (4.4)	5 (7.0)	<0.0001*
Occasionaly	5 (11.1)	23 (32.4)	
Never	5 (11.1)	28 (39.4)	
*Read Meat*			
Daily	2 (4.4)	1 (1.4)	
Weekly once	28 (62.2)	18 (25.4)	
Monthly once	4 (8.9)	11 (15.5)	0.001*
Occasionaly	5 (11.1)	22 (31.0)	
Never	6 (13.3)	19 (26.8)	
*Cauliflower*			
Daily	2 (4.4)	0 (0)	
Weekly once	23 (51.1)	17 (23.9)	
Monthly once	4 (8.9)	13 (18.3)	0.0002*
Occasionaly	5 (11.1)	31 (43.7)	
Never	11 (24.4)	10 (14.1)	
*Greens*			
Daily	21 (46.7)	40 (56.3)	
Weekly once	19 (42.2)	25 (35.2)	
Monthly once	2 (4.4)	1 (1.4)	0.73
Occasionaly	2 (4.4)	4 (5.6)	
Never	1 (2.2)	1 (1.4)	
*Beetroot*			
Daily	0 (0)	2 (2.8)	
Weekly once	16 (35.6)	28 (39.4)	
Monthly once	11 (24.4)	13 (18.3)	0.66
Occasionaly	11 (24.4)	20 (28.2)	
Never	7 (15.6)	8 (11.3)	
*Soyabean*			
Daily	0 (0)	1 (1.4)	
Weekly once	5 (11.1)	10 (14.1)	
Monthly once	5 (11.1)	5 (7.0)	0.27
Occasionaly	20 (44.4)	20 (28.2)	
Never	15 (33.3)	35 (49.3)	

*p < 0.01 denotes statistical significance.

### 3.3 miRNAs profiling among patients with AMI, CAD, and healthy controls

**Figure 1:**
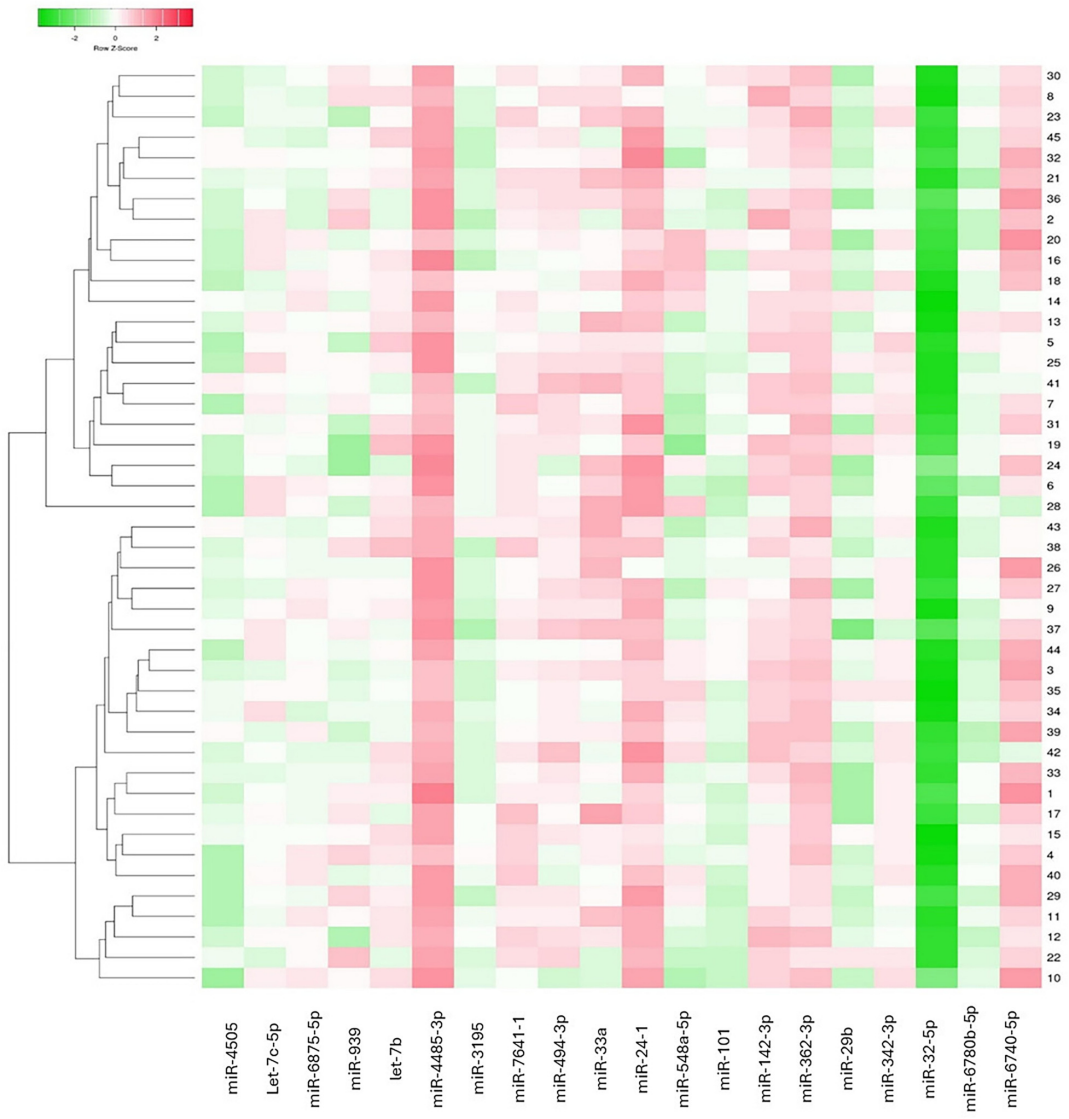
Heatmap shows miRNA expression in AMI patients.

**Figure 2:**
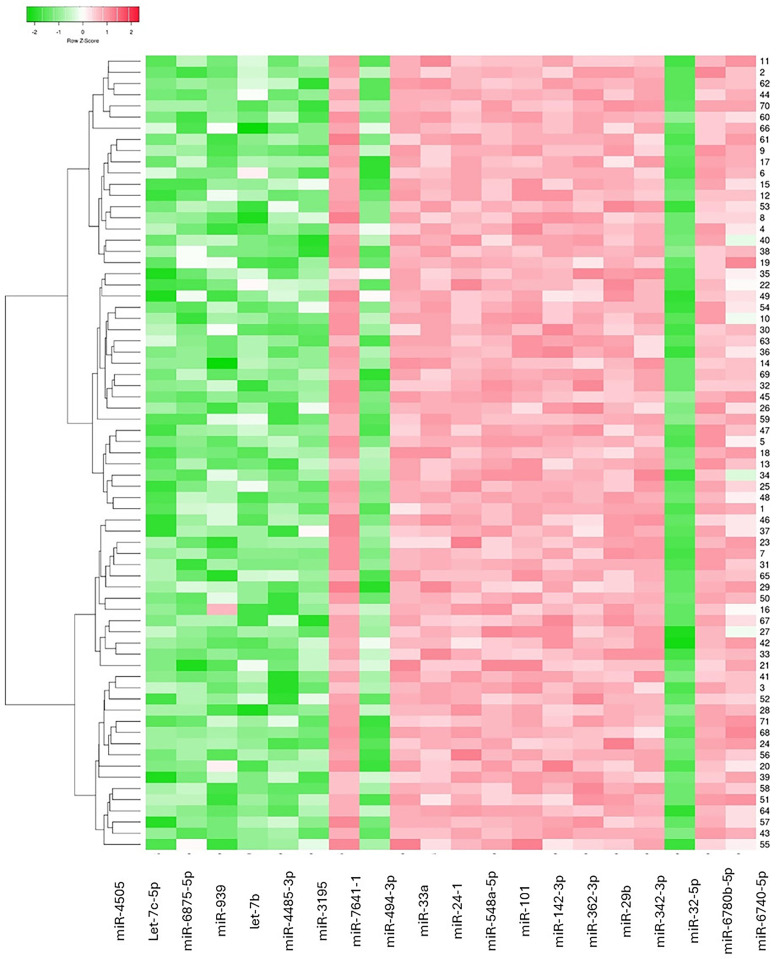
Heatmap shows miRNA expression in CAD patients.

**Figure 3:**
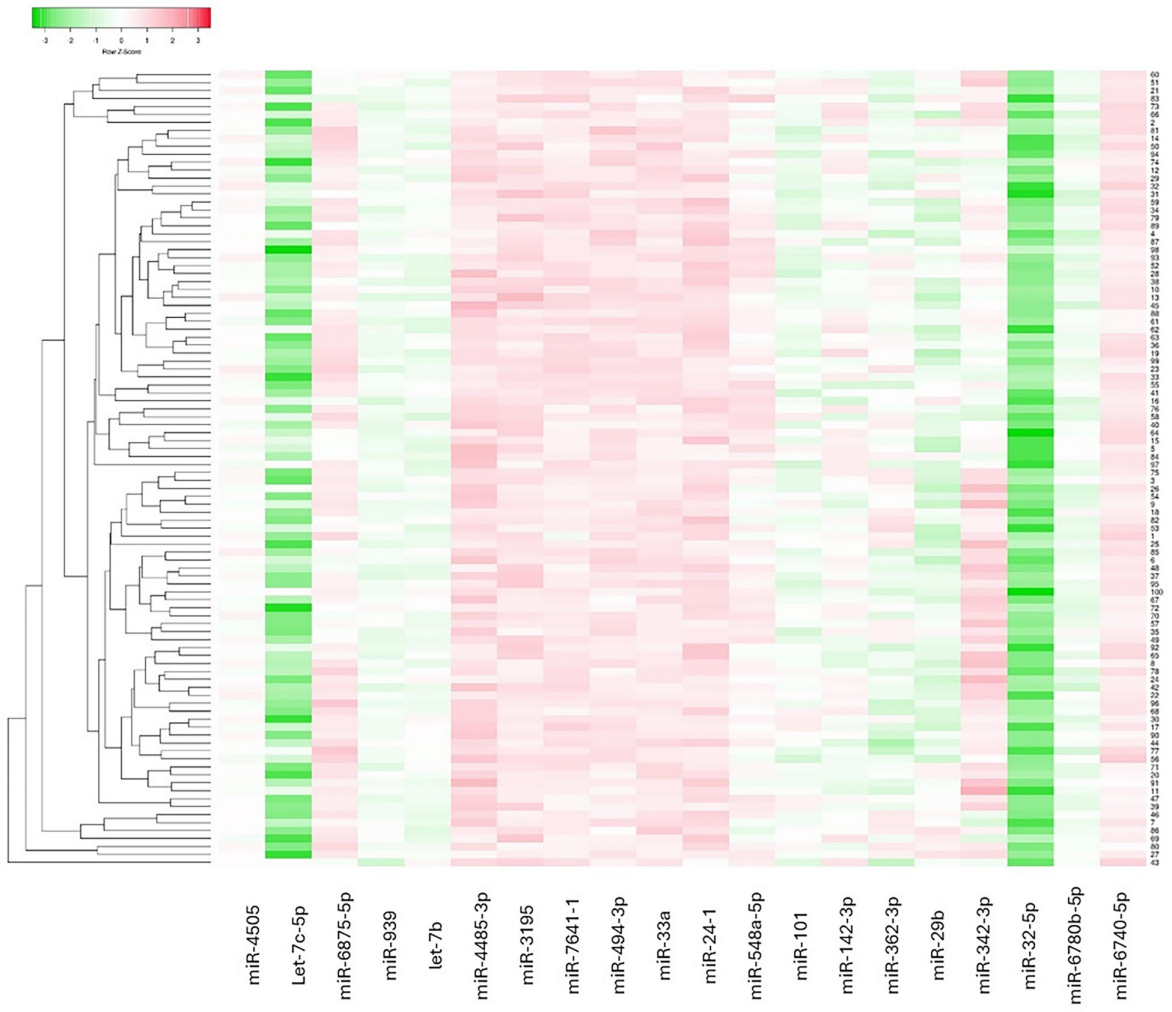
Heatmap shows miRNA expression in healthy controls.

Expression profiling of 20 selected circulating miRNAs was performed across patients diagnosed with AMI, CAD, and HC, with normalization to the reference gene miU6. The expression data were visualized using hierarchical clustering heat maps based on Z-score normalization to identify distinct patterns among the groups (Figures 1–3). In the AMI group, a notable cluster of miRNAs, particularly miR-4485-3p, miR-7641-1, miR-494.3p, miR-let-7b, miR-24-1, and miR-342-3p, demonstrated the upregulation, suggesting their involvement in the acute phase of myocardial injury. The expression pattern in AMI samples was relatively uniform, as shown by the tight clustering and consistent coloration, indicating potential diagnostic markers for AMI. Conversely, other miRNAs were markedly downregulated.

The CAD group exhibited an intermediate profile with modest regulation of most miRNAs and less uniform clustering, likely due to the chronic and stable nature of coronary artery pathology without acute damage. Some miRNAs were observed to be downregulated in AMI but relatively stable or mildly expressed in CAD. The healthy controls displayed a balanced expression profile, with most miRNAs centered near baseline expression levels. However, a few miRNAs showed high expression levels, marking them as potential negative regulators suppressed during myocardial infarction.

### 3.4 Cluster analysis of circulating miRNAs

**Figure 4:**
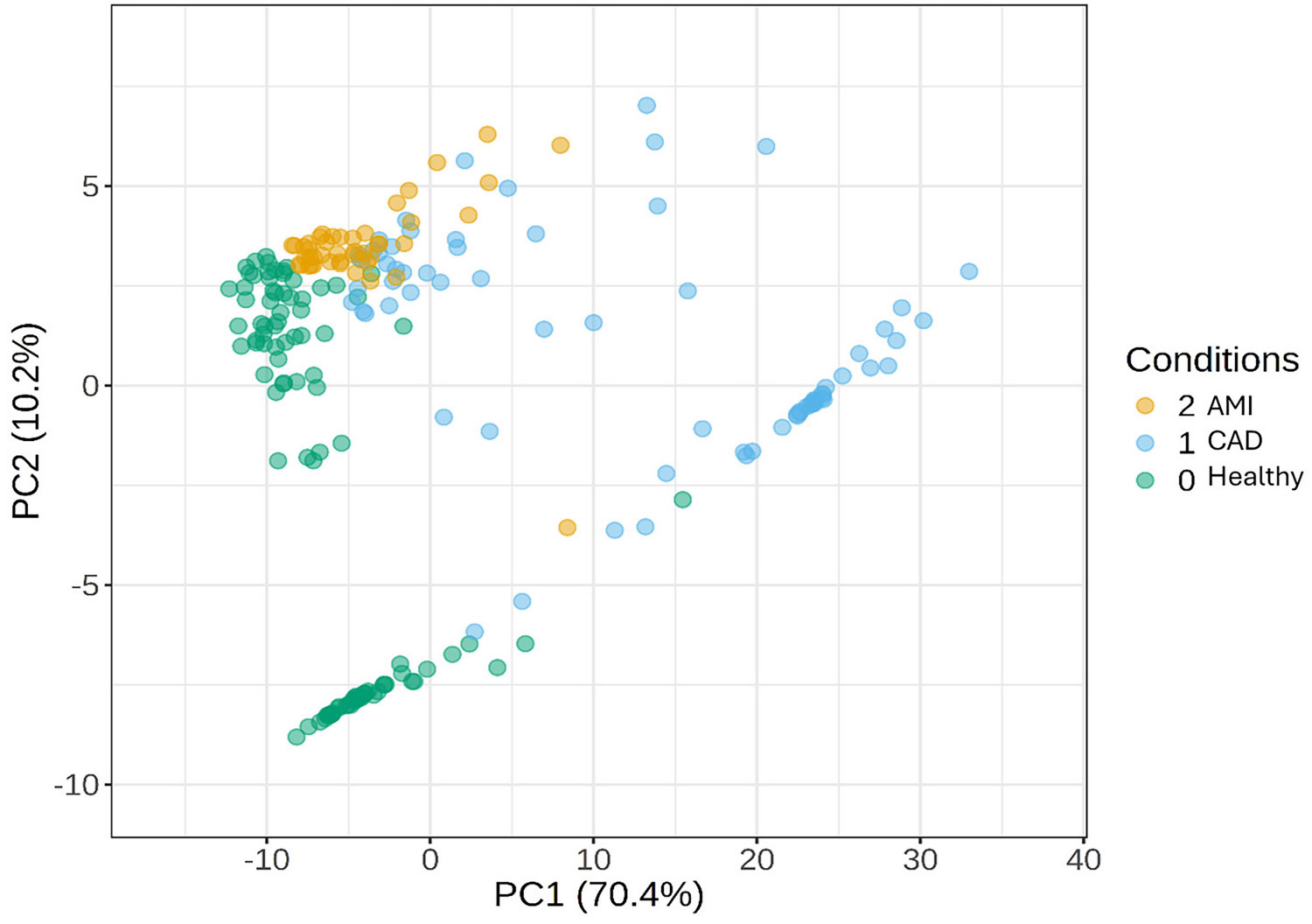
Cluster analysis and principal component analysis (PCA) of circulating miRNAs in AMI, CAD, and healthy controls.

To further explore group-wise segregation, unsupervised clustering of *Δ*Ct values from the 20 profiled miRNAs was performed using k-means clustering (*k* = 3). The resulting cluster map demonstrated a clear separation of AMI cases from CAD and healthy controls, consistent with the expression patterns observed in the heatmaps. Most AMI patients grouped into a distinct cluster, reflecting their unique expression signature, while CAD patients were distributed between AMI and control clusters, indicating intermediate molecular profiles. Healthy controls predominantly clustered together, supporting their relatively stable and baseline expression patterns. Additionally, PCA using normalized *Δ*Ct values further validated this separation. The first two principal components (PC1 = 70.4%, PC2 = 10.2%) captured more than 80% of the variance in the dataset. AMI cases formed a compact and distinct cluster, while CAD patients exhibited greater heterogeneity and overlapped partially with both AMI and control groups. Healthy controls remained tightly clustered and clearly separated from AMI samples. These findings confirm that circulating miRNA profiles provide strong discriminatory power between AMI, CAD, and healthy states (Figure 4).

### 3.5 Differential expression of circulating miRNAs in AMI and CAD patients

A total of 116 participants were analysed, including 45 patients with AMI and 71 patients with CAD. Expression profiles of circulating miRNAs were compared between the two groups using the Kruskal–Walli's rank-sum test. Statistical significance was defined as adjusted *p* < 0.05 after multiple testing correction using Benjamini-Hochberg procedure. The analysis identified a subset of circulating miRNAs that were differentially expressed between groups. Fourteen miRNAs were significantly upregulated in the AMI group, whereas one miRNA was downregulated compared with CAD patients. In contrast, five miRNAs showed no significant difference in expression between the two groups. A detailed summary of group means and test statistics for all miRNAs is presented in Table 4.

**Table 4:** Differential expression of circulating miRNAs in AMI compared with CAD.

Upregulated Delta ct value is higher & adjusted *p*-value is <0.05	Downregulated Delta ct value is lower & adjusted *p*-value is <0.05	Unchanged Adjusted *p* value is >0.05
hsa-miR-4505	hsa-miR-32-5p	hsa-miR-3195
hsa-Let-7c-5p		hsa-miR-548a-5p
hsa-miR-6875-5p		hsa-miR-101
hsa-miR-939		hsa-miR-6780b-5p
hsa-let-7b		hsa-miR-29b
hsa-miR-4485-3p		
hsa-miR-7641-1		
hsa-miR-494-3p		
hsa-miR-33a		
hsa-miR-24-1		
hsa-miR-142-3p		
hsa-miR-362-3p		
hsa-miR-342-3p		
hsa-miR-6740-5p		

### 3.6 Pathway enrichment analysis of upregulated miRNAs

**Figure 5:**
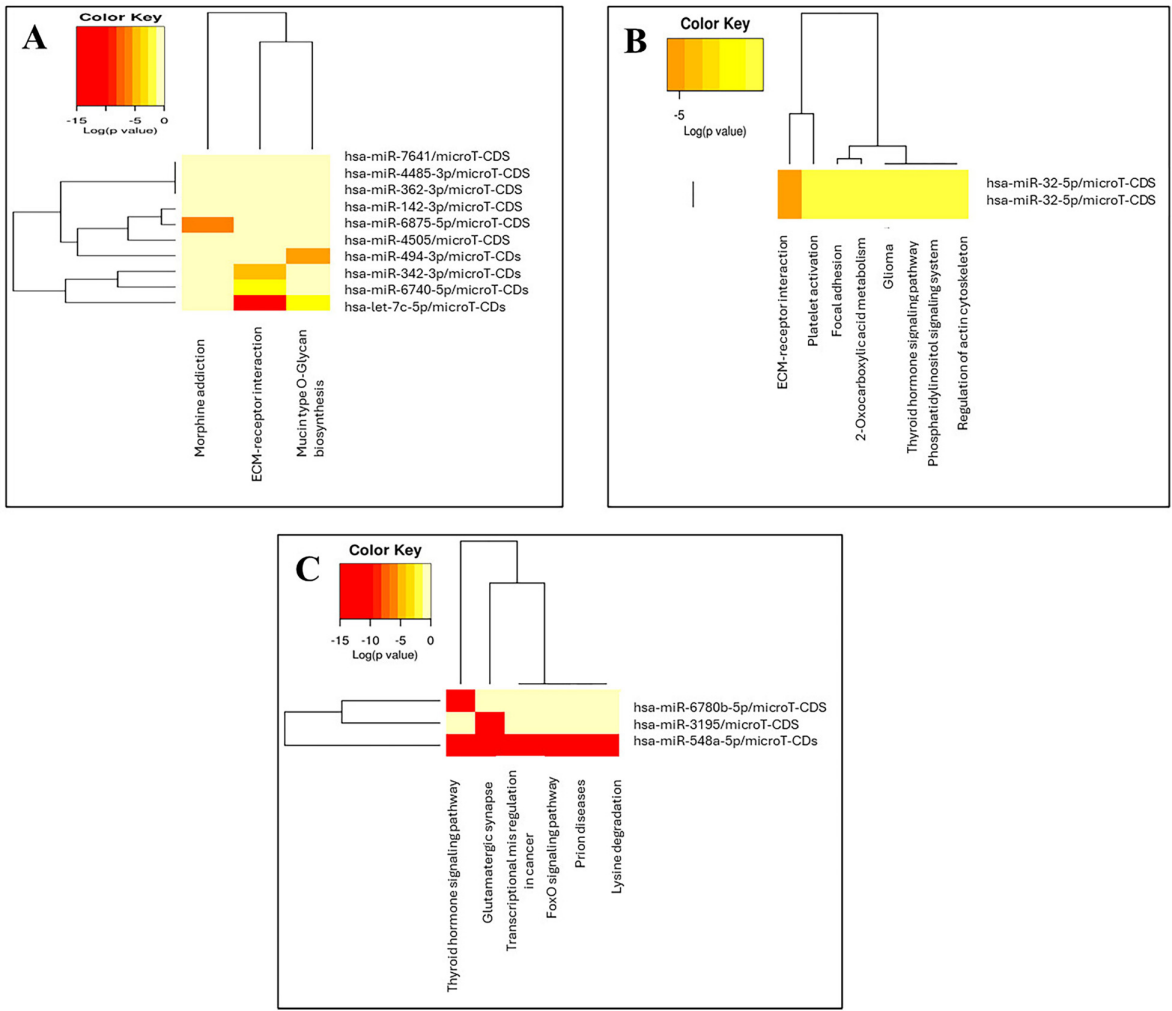
Heat map shows pathway enrichment analysis of miRNAs **(A)** upregulated; **(B)** downregulated; **(C)** unchanged miRNAs.

Pathway enrichment analysis was performed using the panel of upregulated miRNAs identified in AMI patients (Figure 5A). Clustering of predicted target genes revealed significant enrichment in pathways included extracellular matrix (ECM)-receptor interaction, mucin-type O-glycan biosynthesis, and morphine addiction, with adjusted *p*-values below the significance threshold (*p* < 0.05). Notably, ECM-receptor interaction and mucin-type glycan biosynthesis pathways are directly related to processes of cell adhesion, tissue remodeling, and inflammatory signaling, which are central to the pathophysiology of myocardial injury and repair. The enrichment of these pathways supports the functional involvement of the identified miRNAs in cardiac remodeling and vascular pathology following AMI.

### 3.7 Pathway enrichment analysis of downregulated miRNAs

Functional enrichment analysis of the single downregulated miRNA (hsa-miR-32-5p) revealed associations with pathways relevant to cardiovascular pathophysiology, including ECM–receptor interaction, platelet activation, focal adhesion, and regulation of the actin cytoskeleton. These pathways are closely linked to vascular integrity, thrombosis, and cell–matrix communication, processes known to be altered during acute myocardial infarction. The downregulation of hsa-miR-32-5p may therefore contribute to enhanced platelet reactivity and extracellular matrix remodeling, both of which are key events in plaque rupture and thrombus formation (Figure 5B).

### 3.8 Pathway enrichment analysis of unchanged miRNAs

For the miRNAs that did not show significant differential expression between AMI and CAD groups, enrichment analysis identified associations with pathways including steroid hormone signaling, glutamatergic synapse, transcriptional misregulation in cancer, and the FoxO signaling pathway (Figure 5C). However, these associations were less consistent and did not reach the same level of significance as those observed for the upregulated miRNAs. The presence of unchanged miRNAs mapping to diverse signaling and metabolic pathways may reflect background regulatory processes not specifically altered in AMI. This suggests that while these miRNAs participate in general cellular signaling, their expression patterns are not sufficiently modulated to distinguish AMI from CAD in this dataset.

### 3.9 Receiver operating characteristic curve analysis

**Figure 6:**
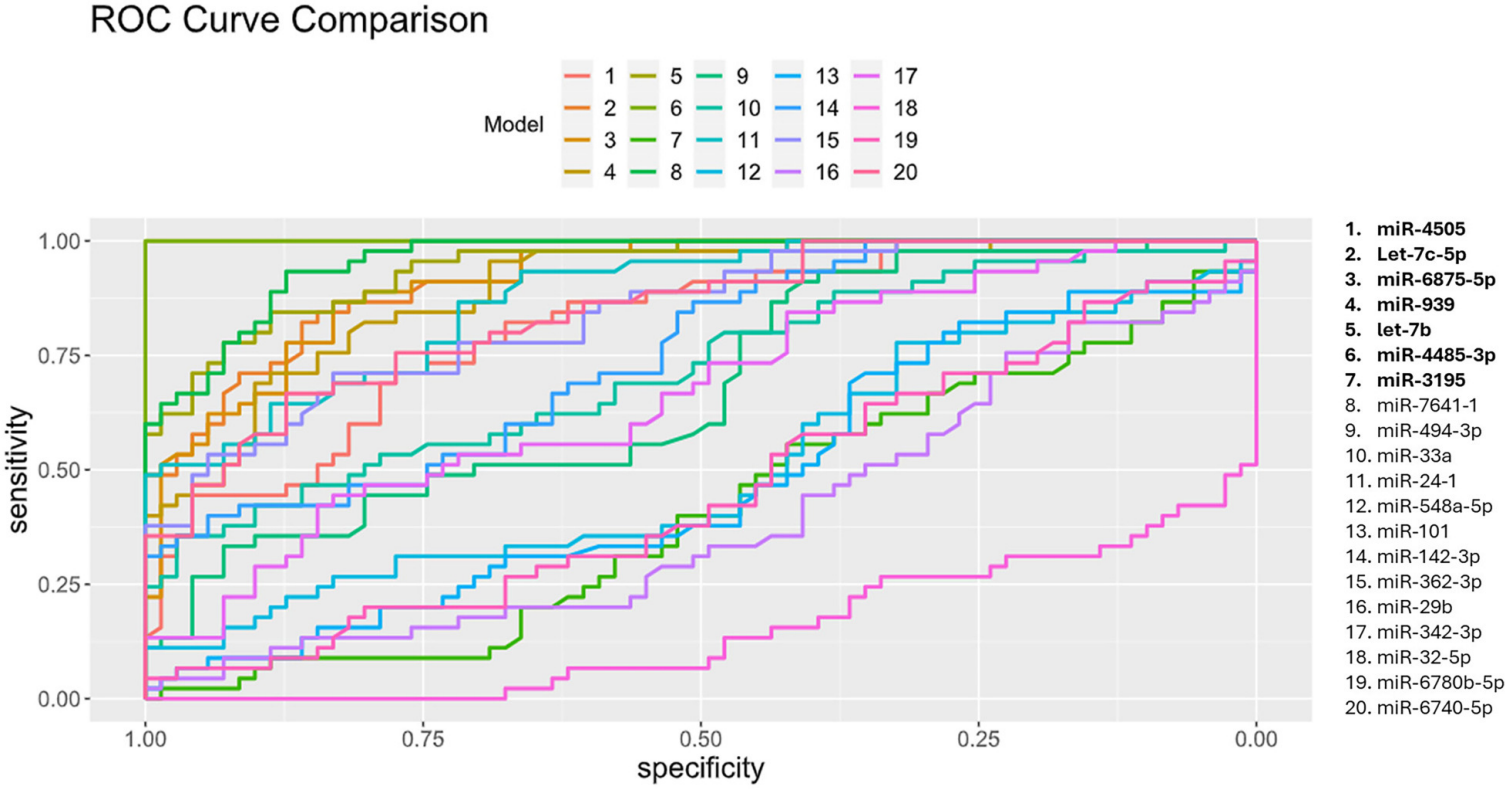
ROC curve comparison analysis of AMI and CAD.

Receiver operating characteristic (ROC) curve analysis was performed to assess the discriminatory power of the selected miRNAs in differentiating AMI patients from CAD patients and healthy controls. Among the tested miRNAs, hsa-miR-4485-3p demonstrated perfect discrimination with an AUC of 1.000 (95% CI: 1.000–1.000, *p* < 0.001). Other highly discriminatory miRNAs included hsa-miR-7641.1 (AUC = 0.961; 95% CI: 0.932–0.989; *p* < 0.001), let-7b (AUC = 0.944; 95% CI: 0.906–0.981; *p* < 0.001), let-7c-5p (AUC = 0.919; 95% CI: 0.871–0.967; *p* < 0.001), and miR-6875-5p (AUC = 0.916; 95% CI: 0.868–0.964; *p* < 0.001). Additionally, miR-939 (AUC = 0.893; 95% CI: 0.835–0.950; *p* < 0.001), miR-24-1 (AUC = 0.877; 95% CI: 0.816–0.937; p < 0.001), miR-362-3p (AUC = 0.841; 95% CI: 0.769–0.912; *p* < 0.001), and miR-6740-5p (AUC = 0.844; 95% CI: 0.774–0.915; *p* < 0.001) also showed good discriminatory performance. Conversely, miR-32-5p had a poor performance with an AUC of 0.149 (95% CI: 0.078–0.220; *p* < 0.001). A comprehensive summary of AUC values, confidence intervals, and pairwise comparisons is provided in Table 5 and Figure 6. The raw data of miRNAs expression can be found in Supplementary Material 1.

**Table 5:** Receiver operating characteristic (ROC) curve analysis of circulating miRNAs distinguishing AMI from CAD.

miRNA	AUC	95% CI	*p*-value
hsa. miR.4505	0.81	0.73–0.89	<0.0001
hsa. Let.7c.5p	0.91	0.87–0.96	<0.0001
hsa. miR.6875.5p	0.91	0.86–0.96	<0.0001
hsa. miR.939	0.89	0.83–0.95	<0.0001
hsa.let.7b	0.94	0.90–0.98	<0.0001
hsa. miR.4485.3p	1.00	1.0–1.0	<0.0001
hsa. miR.3195	0.41	0.30–0.51	0.04
hsa. miR.7641.1	0.96	0.93–0.98	<0.0001
hsa. miR.494.3p	0.67	0.57–0.77	0.0002
hsa. miR.33a	0.71	0.62–0.81	<0.0001
hsa. miR.24.1	0.87	0.81–0.93	<0.0001
hsa. miR.548a.5p	0.50	0.39–0.61	0.45
hsa. miR.101	0.47	0.36–0.58	0.31
hsa. miR.142.3p	0.75	0.66–0.84	<0.0001
hsa. miR.362.3p	0.84	0.76–0.91	<0.0001
hsa. miR.29b	0.39	0.28–0.50	0.02
hsa. miR.342.3p	0.66	0.56–0.76	0.0005
hsa. miR.32.5p	0.14	0.07–0.22	<0.0001
hsa. miR.6780b.5p	0.45	0.34–0.56	0.20
hsa. miR.6740.5p	0.84	0.77–0.91	<0.0001

## 4 Discussion

In this prospective study, we investigated the expression patterns of 20 selected circulating microRNAs (miRNAs) among South Indian patients diagnosed with AMI, stable CAD, and healthy controls, aiming to identify disease-specific signatures that could serve as potential diagnostic biomarkers. Our results revealed a distinct panel of miRNAs that were differentially expressed in AMI patients, notably the upregulation of miR-let-7b, let-7c-5p, miR-362-3p, miR-24-1, and miR-342-3p, alongside significant downregulation of miR-4505, miR-939, miR-4485-3p, and miR-494-3p compared to CAD and healthy controls. These findings underscore the complex pathophysiological processes that characterize acute coronary events, such as heightened inflammatory signaling, endothelial dysfunction, cardiomyocyte apoptosis, and tissue remodeling. The let-7 family, including let-7b and let-7c-5p, has been widely documented to modulate inflammatory and fibrotic responses by targeting interleukin signaling pathways and Toll-like receptors, and their elevation in AMI patients is consistent with an amplified inflammatory milieu in response to ischemic injury, as supported by earlier work (4, 5). Likewise, the increased expression of miR-24-1, which has been shown experimentally to inhibit cardiomyocyte apoptosis and reduce infarct size (6), may represent an endogenous protective response activated during acute injury. The upregulation of miR-342-3p, previously associated with vascular smooth muscle proliferation and vascular remodeling (7), further indicates an active reparative or remodeling process post-infarction. In contrast, the marked downregulation of miR-4485-3p and miR-494-3p in AMI patients is particularly significant given their known roles in promoting cardiomyocyte survival and angiogenesis via PI3K/Akt and Bcl-2 signaling pathways (10). This downregulation may reflect an overwhelmed or impaired protective mechanism in the acute phase, thereby contributing to greater myocardial injury. Similarly, the reduced levels of miR-939, which modulates endothelial function and inflammation (11), could exacerbate endothelial dysfunction and promote plaque instability, key processes in AMI pathogenesis. Notably, CAD patients exhibited intermediate miRNA expression patterns, suggesting a possible continuum of molecular dysregulation from stable atherosclerosis to acute plaque rupture and infarction. For instance, miR-3195 and miR-33a were more highly expressed in CAD patients than in AMI patients or controls, aligning with their recognized roles in lipid metabolism and chronic plaque stabilization (12). Additionally, miRNAs such as miR-142-3p, miR-362-3p, and miR-29b were elevated in both AMI and CAD compared to healthy controls, highlighting their potential involvement in persistent vascular inflammation and remodeling common to both disease states (13). Taking together, these findings suggest that specific miRNAs may act as dynamic molecular markers reflective of disease stage and activity. Clinically, this study supports the utility of circulating miRNAs as minimally invasive biomarkers that could enhance early diagnosis of AMI, especially in South Asian populations known to experience earlier and more severe coronary artery disease (14). Unlike traditional biomarkers like troponin, miRNA quantification with an appropriate endogenous control (15), provide additional information on underlying molecular pathways and may be useful for risk stratification or therapeutic targeting.

ROC analysis demonstrated that several miRNAs, notably miR-4485-3p, let-7b, let-7c-5p, miR-7641-1, and miR-6875-5p, exhibit excellent discriminatory power (AUC >0.9) in differentiating AMI from CAD and controls. Our finding that miR-4485-3p achieved perfect discrimination (AUC = 1.0) is novel, but similar high diagnostic accuracies have been reported for miR-1, miR-208, miR-499, and miR-30a in AMI cohorts (16–18). Schulte & Zeller (2015) highlighted the potential of miRNA-based diagnostics, but few studies demonstrated such strong discriminatory values (13). Our study provides prospective, population-specific evidence that circulating miRNAs may outperform or complement conventional biomarkers like troponin for AMI diagnosis. Taken together, CAD patients exhibited intermediate miRNA expression patterns, suggesting a continuum of molecular dysregulation from stable atherosclerosis to acute plaque rupture and infarction. Clinically, this study supports the utility of circulating miRNAs as minimally invasive biomarkers that could enhance early diagnosis of AMI, especially in South Asian populations known to experience earlier and more severe coronary artery disease (3).

Our finding that let-7 family members (let-7b/let-7c) are altered in AMI is consistent with growing evidence that let-7b-5p is involved in post-injury cardiac remodeling and inflammation. Recent mechanistic studies indicate let-7b (particularly in small extracellular vesicles) modulates cardiac remodeling through innate immune/TLR-related signaling and may affect cardiomyocyte-fibroblast crosstalk, supporting a potential role for circulating let-7b as both a biomarker and a mediator of remodeling after AMI (19). This mechanistic plausibility reinforces our prioritization of let-7 family members in the South-Indian cohort (8). miR-342-3p has been repeatedly implicated in exosome-mediated cardio protection several studies report that exosomal miR-342-3p reduces cardiomyocyte apoptosis and modulates autophagy by directly targeting SOX6 and TFEB. These findings help explain why miR-342-3p shows variable directionality depending on the biofluid (serum/plasma vs. exosomes) and timing (acute vs convalescent) used in different studies: exosome-enriched preparations frequently capture the cardioprotective pool of miR-342-3p that may be depleted or redistributed in whole plasma/serum during the acute phase. This supports our observation that sampling method and time-point can produce discordant miRNA signals and argues for standardized plasma vs exosome protocols in follow-up validations (20). The miR-494 has documented roles in limiting hypoxia/reoxygenation-induced apoptosis and autophagy via modulation of SIRT1 and downstream PI3K/AKT/mTOR signaling. Functional studies in cardiomyocyte models show miR-494 overexpression preserves p-PI3K/p-AKT/p-mTOR signaling and reduces apoptosis, providing a mechanistic basis for our enrichment findings implicating PI3K/AKT/mTOR in the predicted targets of dysregulated miRNAs. These mechanistic data support the biological plausibility that circulating changes in miR-494 reflect cardiomyocyte stress responses and may portend remodeling/repair (21).

Several of the miRNAs that performed well in our ROC/PCA analyses (notably miR-4485-3p, miR-4505, and miR-939) are sparsely represented in the cardiovascular literature. miR-4485 has been characterized mainly in non-cardiac contexts (including as a mitochondrially associated small RNA), and its near-perfect discrimination in our pilot cohort suggests it may either reflect a heart-specific release pattern or a systemic response to acute ischemia; mechanistic follow-up (cellular origin, stability in plasma vs exosomes, and target validation) is therefore a priority (22). Meta-analyses and systematic reviews of circulating miRNAs in ischemic heart disease show that several miRNAs (for example miR-1, miR-21, miR-208, miR-499 and others) have moderate to good diagnostic performance, but heterogeneity in assay type, sample processing and timing limit direct comparisons. Our study advances the field by profiling a prioritized panel in a South Indian prospective cohort and demonstrating strong discriminatory power for specific miRNAs (including some novel signals). Nonetheless, pooling across published studies suggests that panels (multi-miRNA signatures) and harmonized sampling protocols will likely yield the most robust diagnostic tests (23).

Taken together, the mechanistic literature supports the biological relevance of the miRNA families and pathways we identified (let-7 family, miR-342-3p, miR-494 → PI3K/AKT/mTOR, AGE-RAGE and RAAS signaling). We therefore recommend three immediate next steps: validate the top panel (let-7b/c, miR-342-3p, miR-494, miR-4485-3p) in an independent South-Indian cohort with serial sampling (admission, 24 h, 7 d, 30 d) and plasma/exosome fractionation; perform target-level validation (qPCR/protein for prioritized targets such as SIRT1, FOXO1, AGTR1) in matched samples to demonstrate coordinate miRNA–mRNA regulation; and investigate origin of novel miRNAs (e.g., mitochondrial/nuclear) using fractionation and cellular models. These steps will both increase biological confidence and accelerate translation into diagnostics. (This aligns with the priorities suggested by our *in-silico* prioritization work (8).

However, several limitations should be acknowledged: the modest sample size, particularly among AMI cases, may limit statistical power to detect miRNAs with smaller effect sizes; the single-center design and focus on a South Indian population could limit generalizability to other ethnic or regional groups; and the cross-sectional nature of the analysis prevents assessment of temporal miRNA changes before and after infarction. Furthermore, although miU6 was used for normalization, there remains ongoing debate about the most appropriate endogenous controls for plasma miRNA studies (15). Owing to these limitations of sample size we have prioritized miRNAs from public datasets earlier to be studied prospectively in this cohort. Technical limitations include validation of primers for specific miRNA amplification using synthetic RNA standards. Missing data on relevant biomarkers (e.g., Troponin) is also a major limitation of the study. Furthermore, the functional interpretations were primarily based on *in silico* analyses without experimental validation, which restricts mechanistic conclusions regarding the biological roles of the identified miRNAs.

Despite these limitations, the study's strengths include its prospective design, the simultaneous profiling of multiple miRNAs, and the focus on an underrepresented high-risk population. The study also provides an understanding of the targeted miRNA landscape associated with AMI and CAD, particularly in an underrepresented high-risk population. Future directions should include larger multicenter studies to validate these findings, longitudinal sampling to capture dynamic miRNA expression changes following AMI, and mechanistic studies to clarify the functional roles of less characterized miRNAs such as miR-4505 and miR-939. Integrating miRNA expression data with established biomarkers such as troponin may further improve their translational and prognostic utility in cardiovascular disease management, by providing earlier insights on disease pathology or treatment responses.

## 5 Conclusions

This study demonstrates that a defined panel of circulating miRNAs particularly the upregulation of miR-4505, let-7c-5p, miR-939, let-7b, and miR-362-3p, and the concurrent downregulation of miR-4485-3p and miR-494-3p, distinctly characterizes acute myocardial infarction in South Indian patients compared to CAD and Healthy controls. These miRNAs reflect underlying pathophysiological processes such as inflammation, endothelial dysfunction, and impaired cardiomyocyte survival, underscoring their utility as non-invasive biomarkers for early AMI detection. While our findings offer promising diagnostic insights, further validation in larger multi-centre cohorts and longitudinal studies are warranted to confirm their prognostic value and to pave the way for clinical translation.

## Data Availability

The data can be found in the article. The raw miRNA expression dataset is provided in the supplementary materials. Additional data are available from the corresponding author upon reasonable request.

## References

[1] Aboyans V. Global, regional, and national age-sex specific all-cause and cause-specific mortality for 240 causes of death, 1990–2013: a systematic analysis for the global burden of disease study 2013. Lancet. (2015) 385(9963):117–71. 10.1016/S0140-6736(14)61682-2 25530442 PMC4340604

[2] Martin SS Aday AW Almarzooq ZI Anderson CA Arora P Avery CL. Comm.

[3] Ajay VS Prabhakaran D. Coronary heart disease in Indians: implications of the INTERHEART study. Indian J Med Res. (2010) 132(5):561–6. 10.4103/0971-5916.73396 21150008 PMC3028954

[4] Boon RA Dimmeler S. MicroRNAs in myocardial infarction. Nat Rev Cardiol. (2015) 12(3):135–42. 10.1038/nrcardio.2014.207 25511085

[5] Raso A Dirkx E Philippen LE Maiellaro I Regan RF Coumans WA. Therapeutic inhibition of miR-15 in myocardial infarction-induced heart failure. EBioMedicine. (2018) 32:100–14. 10.1016/j.ebiom.2018.05.009

[6] Qian L Van Laake LW Huang Y Liu S Wendland MF Srivastava D. miR-24 inhibits apoptosis and represses bim in mouse cardiomyocytes. J Exp Med. (2011) 208(3):549–60. 10.1084/jem.20101821 21383058 PMC3058576

[7] Xu T Liu J Li Z Jiang L Wang G Li W. MicroRNA-342-3p promotes vascular smooth muscle cell proliferation and neointimal hyperplasia after vascular injury. J Vasc Res. (2017) 54(1):1–11. 10.1159/000452264

[8] Venugopal P George M Kandadai SD Balakrishnan K Uppugunduri CR. Prioritization of microRNA biomarkers for a prospective evaluation in a cohort of myocardial infarction patients based on their mechanistic role using public datasets. Front Cardiovasc Med. (2022) 9:981335. 10.3389/fcvm.2022.981335 36407428 PMC9668885

[9] Trakunram K Champoochana N Chaniad P Thongsuksai P Raungrut P. MicroRNA isolation by trizol-based method and its stability in stored serum and cDNA derivatives. Asian Pac J Cancer Prev. (2019) 20(6):1641–7. 10.31557/APJCP.2019.20.6.1641 31244282 PMC7021594

[10] Zhao Y Ransom JF Li A Vedantham V von Drehle M Muth AN. Dysregulation of cardiogenesis, cardiac conduction, and cell cycle in mice lacking miRNA-1-2. Cell. (2010) 129(2):303–17. 10.1016/j.cell.2007.03.030 17397913

[11] Zhou Y Li W Li Y Xu J Wang H. MicroRNA-939 inhibits endothelial cell proliferation and migration by targeting nitric oxide synthase. Cell Physiol Biochem. (2017) 43(4):1670–80. 10.1159/000484029

[12] Rayner KJ Suarez Y Davalos A Parathath S Fitzgerald ML Tamehiro N. MiR-33 contributes to the regulation of cholesterol homeostasis. Science. (2011) 328(5985):1570–3. 10.1126/science.1189862 PMC3114628 20466885

[13] Schulte C Zeller T. microRNA-based diagnostics and therapy in cardiovascular disease—summing up the facts. Cardiovasc Diagn Ther. (2015) 5(1):17–36. 10.3978/j.issn.2223-3652.2015.01.02 25774345 PMC4329169

[14] Enas EA Dhawan J Petkar S. Coronary artery disease in Asian Indians: lessons learnt and the role of lipoprotein(a). Indian Heart J. (1997) 49(1):25–34.PMID: 9130422 9130422

[15] Pritchard CC Kroh E Wood B Arroyo JD Dougherty KJ Miyaji MM. Blood cell origin of circulating microRNAs: a cautionary note for cancer biomarker studies. Cancer Prev Res. (2012) 5(3):492–7. 10.1158/1940-6207.CAPR-11-0370 PMC4186243 22158052

[16] Widera C Gupta SK Lorenzen JM Bang C Bauersachs J Bethmann K. Diagnostic and prognostic impact of six circulating microRNAs in acute coronary syndrome. J Mol Cell Cardiol. (2011) 51(5):872–5. 10.1016/j.yjmcc.2011.07.011 21806992

[17] Gidlöf O Smith JG Miyazu K Gilje P Spencer A Blomquist S. Circulating cardio-enriched microRNAs are associated with long-term prognosis following myocardial infarction. BMC Cardiovasc Disord. (2013) 13:12. 10.1186/1471-2261-13-12 23448306 PMC3598930

[18] Li YQ Zhang MF Wen HY Hu CL Liu R Wei HY. Comparing the diagnostic values of circulating microRNAs and cardiac troponin T in patients with acute myocardial infarction. Clin Biochem. (2013) 46(7-8):804–8. 10.1016/j.clinbiochem.2013.02.011 PMC3552456 23420161

[19] Zhang Y Cui H Zhao M Yu H Xu W Wang Z. Cardiomyocyte-derived small extracellular vesicle-transported let-7b-5p modulates cardiac remodeling via TLR7 signaling pathway. FASEB J. (2024 Nov 30) 38(22):e70196. 10.1096/fj.202302587RRR 39570019

[20] Wang B Cao C Han D Bai J Guo J Guo Q. Dysregulation of miR-342-3p in plasma exosomes derived from convalescent AMI patients and its consequences on cardiac repair. Biomed Pharmacother. (2021) 142:112056. 10.1016/j.biopha.2021.112056 34435593

[21] Ning S Li Z Ji Z Fan D Wang K Wang Q. MicroRNA-494 suppresses hypoxia/reoxygenation-induced cardiomyocyte apoptosis and autophagy via the PI3K/AKT/mTOR signaling pathway by targeting SIRT1. Mol Med Rep. (2020) 22(6):5231–42. 10.3892/mmr.2020.11636 33174056 PMC7646990

[22] Farfán N Sanhueza N Briones M Burzio LO Burzio VA. Antisense noncoding mitochondrial RNA-2 gives rise to miR-4485-3p by dicer processing *in vitro*. Biol Res. (2021) 54(1):33. 10.1186/s40659-021-00356-0 34666824 PMC8527801

[23] Alcibahy Y Darwish R Abu-Sharia G Maes Q Elgamassy O. Circulating microRNAs as biomarkers for ischemic heart disease: a systematic review and gene set enrichment analysis. Front Med. (2025) 12:1545023. 10.3389/fmed.2025.1545023 PMC12411434 40917857

