# Supplementary material for: Utility of miRNA biomarkers in patients with acute myocardial infarction (UNIAMI)—results from a prospective pilot study
Source: Front Cardiovasc Med. 2026 Jun 11;13:1730999. doi: 10.3389/fcvm.2026.1730999 (PMC13294383; doi:10.3389/fcvm.2026.1730999)
Supplement: Supplementary file 1 [file datasheet1.pdf]

Table S1: ROC Curve Areas, 0.95 Level Confidence Intervals, and Tests of Area>0.5						
Curve	Variable	AUC	AUC_lower	AUC_upper	Z	p.value
**1**	hsa.miR.4505	0.8160	0.7393	0.8926	8.0826	3.3307e-16 ***
**2**	hsa.Let.7c.5p	0.9188	0.8711	0.9665	17.2043	0.0000e+00 ***
**3**	hsa.miR.6875.5p	0.9156	0.8675	0.9638	16.9165	0.0000e+00 ***
**4**	hsa.miR.939	0.8926	0.8350	0.9503	13.3504	0.0000e+00 ***
**5**	hsa.let.7b	0.9437	0.9060	0.9813	23.1187	0.0000e+00 ***
**6**	hsa.miR.4485.3p	1.0000	1.0000	1.0000	Inf	0.0000e+00 ***
**7**	hsa.miR.3195	0.4110	0.3054	0.5165	-1.6537	4.9089e-02 *
**8**	hsa.miR.7641.1	0.9606	0.9319	0.9892	31.5205	0.0000e+00 ***
**9**	hsa.miR.494.3p	0.6782	0.5793	0.7772	3.5307	2.0724e-04 ***
**10**	hsa.miR.33a	0.7188	0.6219	0.8157	4.4261	4.7968e-06 ***
**11**	hsa.miR.24.1	0.8768	0.8163	0.9374	12.2044	0.0000e+00 ***
**12**	hsa.miR.548a.5p	0.5061	0.3943	0.6179	0.1070	4.5739e-01
**13**	hsa.miR.101	0.4732	0.3644	0.5821	-0.4818	3.1498e-01
**14**	hsa.miR.142.3p	0.7526	0.6647	0.8404	5.6354	8.7340e-09 ***
**15**	hsa.miR.362.3p	0.8405	0.7689	0.9121	9.3205	0.0000e+00 ***
**16**	hsa.miR.29b	0.3944	0.2875	0.5013	-1.9370	2.6375e-02 *

Table S1: ROC Curve Areas, 0.95 Level Confidence Intervals, and Tests of Area>0.5						
Curve	Variable	AUC	AUC_lower	AUC_upper	Z	p.value
**17**	hsa.miR.342.3p	0.6690	0.5688	0.7692	3.3060	4.7327e-04 ***
**18**	hsa.miR.32.5p	0.1490	0.0778	0.2201	-9.6690	0.0000e+00 ***
**19**	hsa.miR.6780b.5p	0.4537	0.3454	0.5620	-0.8381	2.0097e-01
**20**	hsa.miR.6740.5p	0.8443	0.7736	0.9150	9.5495	0.0000e+00 ***
Note:						
Signif. codes: 0 ' *** ' 0.001 ' ** ' 0.01 ' * ' 0.05 '.' 0.1 ' ' 1						

Table S2: Pairwise Comparisons of ROC Curve Areas and 0.95 Level Confidence Intervals								
Variable1	AUC1	Variable2	AUC2	Z	p.value	AUC_diff	AUC_diff_lower	AUC_diff_upper
hsa.miR.4505	0.8160	hsa.Let.7c.5p	0.9188	-3.4367	5.8887e-04 ***	-0.1028	-0.1615	-0.0442
hsa.miR.4505	0.8160	hsa.miR.6875.5p	0.9156	-3.1595	1.5804e-03 **	-0.0997	-0.1615	-0.0378
hsa.miR.4505	0.8160	hsa.miR.939	0.8926	-2.1039	3.5383e-02 *	-0.0767	-0.1481	-0.0052
hsa.miR.4505	0.8160	hsa.let.7b	0.9437	-3.8541	1.1617e-04 ***	-0.1277	-0.1926	-0.0628
hsa.miR.4505	0.8160	hsa.miR.4485.3p	1.0000	-4.7078	2.5038e-06 ***	-0.1840	-0.2607	-0.1074
hsa.miR.4505	0.8160	hsa.miR.3195	0.4110	10.5146	7.3977e-26 ***	0.4050	0.3295	0.4805
hsa.miR.4505	0.8160	hsa.miR.7641.1	0.9606	-4.4587	8.2472e-06 ***	-0.1446	-0.2082	-0.0810

Table S2: Pairwise Comparisons of ROC Curve Areas and 0.95 Level Confidence Intervals								
Variable1	AUC1	Variable2	AUC2	Z	p.value	AUC_diff	AUC_diff_lower	AUC_diff_upper
hsa.miR.4505	0.8160	hsa.miR.494.3p	0.6782	4.2286	2.3515e-05 ***	0.1377	0.0739	0.2015
hsa.miR.4505	0.8160	hsa.miR.33a	0.7188	2.9073	3.6457e-03 **	0.0972	0.0317	0.1627
hsa.miR.4505	0.8160	hsa.miR.24.1	0.8768	-1.8772	6.0487e-02 .	-0.0609	-0.1244	0.0027
hsa.miR.4505	0.8160	hsa.miR.548a.5p	0.5061	7.0430	1.8816e-12 ***	0.3099	0.2236	0.3961
hsa.miR.4505	0.8160	hsa.miR.101	0.4732	9.3638	7.6890e-21 ***	0.3427	0.2710	0.4145
hsa.miR.4505	0.8160	hsa.miR.142.3p	0.7526	1.9885	4.6754e-02 *	0.0634	0.0009	0.1259
hsa.miR.4505	0.8160	hsa.miR.362.3p	0.8405	-0.9791	3.2754e-01	-0.0246	-0.0738	0.0246
hsa.miR.4505	0.8160	hsa.miR.29b	0.3944	9.5845	9.2863e-22 ***	0.4216	0.3354	0.5078
hsa.miR.4505	0.8160	hsa.miR.342.3p	0.6690	4.4099	1.0340e-05 ***	0.1469	0.0816	0.2123
hsa.miR.4505	0.8160	hsa.miR.32.5p	0.1490	17.1046	1.3721e-65 ***	0.6670	0.5906	0.7434
hsa.miR.4505	0.8160	hsa.miR.6780b.5p	0.4537	9.1854	4.1018e-20 ***	0.3623	0.2850	0.4396
hsa.miR.4505	0.8160	hsa.miR.6740.5p	0.8443	-0.8227	4.1067e-01	-0.0283	-0.0958	0.0392
hsa.Let.7c.5p	0.9188	hsa.miR.6875.5p	0.9156	0.1438	8.8564e-01	0.0031	-0.0395	0.0458
hsa.Let.7c.5p	0.9188	hsa.miR.939	0.8926	0.8903	3.7329e-01	0.0261	-0.0314	0.0837
hsa.Let.7c.5p	0.9188	hsa.let.7b	0.9437	-1.3702	1.7064e-01	-0.0249	-0.0605	0.0107
hsa.Let.7c.5p	0.9188	hsa.miR.4485.3p	1.0000	-3.3367	8.4777e-04 ***	-0.0812	-0.1289	-0.0335

Table S2: Pairwise Comparisons of ROC Curve Areas and 0.95 Level Confidence Intervals								
Variable1	AUC1	Variable2	AUC2	Z	p.value	AUC_diff	AUC_diff_lower	AUC_diff_upper
hsa.Let.7c.5p	0.9188	hsa.miR.3195	0.4110	12.0847	1.2725e-33 ***	0.5078	0.4255	0.5902
hsa.Let.7c.5p	0.9188	hsa.miR.7641.1	0.9606	-2.0522	4.0148e-02 *	-0.0418	-0.0817	-0.0019
hsa.Let.7c.5p	0.9188	hsa.miR.494.3p	0.6782	6.0604	1.3580e-09 ***	0.2405	0.1627	0.3183
hsa.Let.7c.5p	0.9188	hsa.miR.33a	0.7188	5.3527	8.6674e-08 ***	0.2000	0.1268	0.2732
hsa.Let.7c.5p	0.9188	hsa.miR.24.1	0.8768	1.7969	7.2353e-02 .	0.0419	-0.0038	0.0877
hsa.Let.7c.5p	0.9188	hsa.miR.548a.5p	0.5061	8.7966	1.4101e-18 ***	0.4127	0.3207	0.5046
hsa.Let.7c.5p	0.9188	hsa.miR.101	0.4732	10.1551	3.1439e-24 ***	0.4455	0.3595	0.5315
hsa.Let.7c.5p	0.9188	hsa.miR.142.3p	0.7526	4.8765	1.0796e-06 ***	0.1662	0.0994	0.2330
hsa.Let.7c.5p	0.9188	hsa.miR.362.3p	0.8405	3.0877	2.0169e-03 **	0.0782	0.0286	0.1279
hsa.Let.7c.5p	0.9188	hsa.miR.29b	0.3944	11.4901	1.4797e-30 ***	0.5244	0.4350	0.6139
hsa.Let.7c.5p	0.9188	hsa.miR.342.3p	0.6690	6.7609	1.3710e-11 ***	0.2498	0.1774	0.3222
hsa.Let.7c.5p	0.9188	hsa.miR.32.5p	0.1490	22.7935	5.3235e-115 ***	0.7698	0.7036	0.8360
hsa.Let.7c.5p	0.9188	hsa.miR.6780b.5p	0.4537	10.6445	1.8496e-26 ***	0.4651	0.3795	0.5507
hsa.Let.7c.5p	0.9188	hsa.miR.6740.5p	0.8443	2.6483	8.0904e-03 **	0.0745	0.0194	0.1296
hsa.miR.6875.5p	0.9156	hsa.miR.939	0.8926	0.8099	4.1801e-01	0.0230	-0.0327	0.0787
hsa.miR.6875.5p	0.9156	hsa.let.7b	0.9437	-1.5206	1.2836e-01	-0.0280	-0.0641	0.0081

Table S2: Pairwise Comparisons of ROC Curve Areas and 0.95 Level Confidence Intervals								
Variable1	AUC1	Variable2	AUC2	Z	p.value	AUC_diff	AUC_diff_lower	AUC_diff_upper
hsa.miR.6875.5p	0.9156	hsa.miR.4485.3p	1.0000	-3.4330	5.9701e-04 ***	-0.0844	-0.1325	-0.0362
hsa.miR.6875.5p	0.9156	hsa.miR.3195	0.4110	11.9659	5.3603e-33 ***	0.5047	0.4220	0.5874
hsa.miR.6875.5p	0.9156	hsa.miR.7641.1	0.9606	-2.3634	1.8110e-02 *	-0.0449	-0.0822	-0.0077
hsa.miR.6875.5p	0.9156	hsa.miR.494.3p	0.6782	5.9661	2.4304e-09 ***	0.2374	0.1594	0.3154
hsa.miR.6875.5p	0.9156	hsa.miR.33a	0.7188	5.2279	1.7148e-07 ***	0.1969	0.1231	0.2707
hsa.miR.6875.5p	0.9156	hsa.miR.24.1	0.8768	1.7007	8.8994e-02 .	0.0388	-0.0059	0.0835
hsa.miR.6875.5p	0.9156	hsa.miR.548a.5p	0.5061	8.4231	3.6662e-17 ***	0.4095	0.3142	0.5048
hsa.miR.6875.5p	0.9156	hsa.miR.101	0.4732	9.9863	1.7500e-23 ***	0.4424	0.3556	0.5292
hsa.miR.6875.5p	0.9156	hsa.miR.142.3p	0.7526	4.8036	1.5584e-06 ***	0.1631	0.0965	0.2296
hsa.miR.6875.5p	0.9156	hsa.miR.362.3p	0.8405	3.1242	1.7829e-03 **	0.0751	0.0280	0.1222
hsa.miR.6875.5p	0.9156	hsa.miR.29b	0.3944	11.1648	6.0598e-29 ***	0.5213	0.4298	0.6128
hsa.miR.6875.5p	0.9156	hsa.miR.342.3p	0.6690	6.7331	1.6607e-11 ***	0.2466	0.1748	0.3184
hsa.miR.6875.5p	0.9156	hsa.miR.32.5p	0.1490	22.0479	1.0001e-107 ***	0.7667	0.6985	0.8348
hsa.miR.6875.5p	0.9156	hsa.miR.6780b.5p	0.4537	10.4740	1.1371e-25 ***	0.4620	0.3755	0.5484
hsa.miR.6875.5p	0.9156	hsa.miR.6740.5p	0.8443	2.6082	9.1033e-03 **	0.0714	0.0177	0.1250
hsa.miR.939	0.8926	hsa.let.7b	0.9437	-1.9250	5.4232e-02 .	-0.0510	-0.1030	0.0009

**Table S2: Pairwise Comparisons of ROC Curve Areas and 0.95 Level Confidence Intervals**								
Variable1	AUC1	Variable2	AUC2	Z	p.value	AUC_diff	AUC_diff_lower	AUC_diff_upper
hsa.miR.939	0.8926	hsa.miR.4485.3p	1.0000	-3.6502	2.6202e-04 ***	-0.1074	-0.1650	-0.0497
hsa.miR.939	0.8926	hsa.miR.3195	0.4110	10.6693	1.4173e-26 ***	0.4817	0.3932	0.5702
hsa.miR.939	0.8926	hsa.miR.7641.1	0.9606	-2.4661	1.3659e-02 *	-0.0679	-0.1219	-0.0139
hsa.miR.939	0.8926	hsa.miR.494.3p	0.6782	5.1347	2.8252e-07 ***	0.2144	0.1326	0.2962
hsa.miR.939	0.8926	hsa.miR.33a	0.7188	4.2578	2.0646e-05 ***	0.1739	0.0938	0.2539
hsa.miR.939	0.8926	hsa.miR.24.1	0.8768	0.5375	5.9091e-01	0.0158	-0.0418	0.0734
hsa.miR.939	0.8926	hsa.miR.548a.5p	0.5061	7.5740	3.6199e-14 ***	0.3865	0.2865	0.4866
hsa.miR.939	0.8926	hsa.miR.101	0.4732	9.0977	9.2273e-20 ***	0.4194	0.3291	0.5098
hsa.miR.939	0.8926	hsa.miR.142.3p	0.7526	3.3510	8.0532e-04 ***	0.1401	0.0581	0.2220
hsa.miR.939	0.8926	hsa.miR.362.3p	0.8405	1.5820	1.1366e-01	0.0521	-0.0125	0.1167
hsa.miR.939	0.8926	hsa.miR.29b	0.3944	10.1888	2.2247e-24 ***	0.4983	0.4024	0.5941
hsa.miR.939	0.8926	hsa.miR.342.3p	0.6690	5.3773	7.5607e-08 ***	0.2236	0.1421	0.3051
hsa.miR.939	0.8926	hsa.miR.32.5p	0.1490	18.7253	3.0791e-78 ***	0.7437	0.6658	0.8215
hsa.miR.939	0.8926	hsa.miR.6780b.5p	0.4537	9.5042	2.0159e-21 ***	0.4390	0.3484	0.5295
hsa.miR.939	0.8926	hsa.miR.6740.5p	0.8443	1.4332	1.5180e-01	0.0484	-0.0178	0.1145
hsa.let.7b	0.9437	hsa.miR.4485.3p	1.0000	-2.9357	3.3279e-03 **	-0.0563	-0.0940	-0.0187

Table S2: Pairwise Comparisons of ROC Curve Areas and 0.95 Level Confidence Intervals								
Variable1	AUC1	Variable2	AUC2	Z	p.value	AUC_diff	AUC_diff_lower	AUC_diff_upper
hsa.let.7b	0.9437	hsa.miR.3195	0.4110	12.0730	1.4672e-33 ***	0.5327	0.4462	0.6192
hsa.let.7b	0.9437	hsa.miR.7641.1	0.9606	-1.1166	2.6417e-01	-0.0169	-0.0466	0.0128
hsa.let.7b	0.9437	hsa.miR.494.3p	0.6782	6.4117	1.4387e-10 ***	0.2654	0.1843	0.3465
hsa.let.7b	0.9437	hsa.miR.33a	0.7188	5.7184	1.0751e-08 ***	0.2249	0.1478	0.3020
hsa.let.7b	0.9437	hsa.miR.24.1	0.8768	2.9472	3.2064e-03 **	0.0668	0.0224	0.1113
hsa.let.7b	0.9437	hsa.miR.548a.5p	0.5061	8.6739	4.1772e-18 ***	0.4376	0.3387	0.5364
hsa.let.7b	0.9437	hsa.miR.101	0.4732	10.0892	6.1670e-24 ***	0.4704	0.3790	0.5618
hsa.let.7b	0.9437	hsa.miR.142.3p	0.7526	5.2845	1.2606e-07 ***	0.1911	0.1202	0.2619
hsa.let.7b	0.9437	hsa.miR.362.3p	0.8405	4.0847	4.4131e-05 ***	0.1031	0.0536	0.1526
hsa.let.7b	0.9437	hsa.miR.29b	0.3944	11.3569	6.8540e-30 ***	0.5493	0.4545	0.6441
hsa.let.7b	0.9437	hsa.miR.342.3p	0.6690	6.8802	5.9776e-12 ***	0.2746	0.1964	0.3529
hsa.let.7b	0.9437	hsa.miR.32.5p	0.1490	23.3574	1.1595e-120 ***	0.7947	0.7280	0.8614
hsa.let.7b	0.9437	hsa.miR.6780b.5p	0.4537	10.6792	1.2741e-26 ***	0.4900	0.4001	0.5799
hsa.let.7b	0.9437	hsa.miR.6740.5p	0.8443	3.2448	1.1752e-03 **	0.0994	0.0393	0.1594
hsa.miR.4485.3p	1.0000	hsa.miR.3195	0.4110	10.9397	7.4426e-28 ***	0.5890	0.4835	0.6946
hsa.miR.4485.3p	1.0000	hsa.miR.7641.1	0.9606	2.6990	6.9548e-03 **	0.0394	0.0108	0.0681

Table S2: Pairwise Comparisons of ROC Curve Areas and 0.95 Level Confidence Intervals								
Variable1	AUC1	Variable2	AUC2	Z	p.value	AUC_diff	AUC_diff_lower	AUC_diff_upper
hsa.miR.4485.3p	1.0000	hsa.miR.494.3p	0.6782	6.3732	1.8510e-10 ***	0.3218	0.2228	0.4207
hsa.miR.4485.3p	1.0000	hsa.miR.33a	0.7188	5.6894	1.2750e-08 ***	0.2812	0.1843	0.3781
hsa.miR.4485.3p	1.0000	hsa.miR.24.1	0.8768	3.9887	6.6430e-05 ***	0.1232	0.0626	0.1837
hsa.miR.4485.3p	1.0000	hsa.miR.548a.5p	0.5061	8.6595	4.7379e-18 ***	0.4939	0.3821	0.6057
hsa.miR.4485.3p	1.0000	hsa.miR.101	0.4732	9.4836	2.4555e-21 ***	0.5268	0.4179	0.6356
hsa.miR.4485.3p	1.0000	hsa.miR.142.3p	0.7526	5.5202	3.3871e-08 ***	0.2474	0.1596	0.3353
hsa.miR.4485.3p	1.0000	hsa.miR.362.3p	0.8405	4.3647	1.2729e-05 ***	0.1595	0.0879	0.2311
hsa.miR.4485.3p	1.0000	hsa.miR.29b	0.3944	11.1053	1.1829e-28 ***	0.6056	0.4987	0.7125
hsa.miR.4485.3p	1.0000	hsa.miR.342.3p	0.6690	6.4742	9.5343e-11 ***	0.3310	0.2308	0.4312
hsa.miR.4485.3p	1.0000	hsa.miR.32.5p	0.1490	23.4417	1.6055e-121 ***	0.8510	0.7799	0.9222
hsa.miR.4485.3p	1.0000	hsa.miR.6780b.5p	0.4537	9.8850	4.8355e-23 ***	0.5463	0.4380	0.6546
hsa.miR.4485.3p	1.0000	hsa.miR.6740.5p	0.8443	4.3190	1.5675e-05 ***	0.1557	0.0850	0.2264
hsa.miR.3195	0.4110	hsa.miR.7641.1	0.9606	-11.7030	1.2300e-31 ***	-0.5496	-0.6417	-0.4576
hsa.miR.3195	0.4110	hsa.miR.494.3p	0.6782	-8.7264	2.6295e-18 ***	-0.2673	-0.3273	-0.2073
hsa.miR.3195	0.4110	hsa.miR.33a	0.7188	-10.0649	7.8956e-24 ***	-0.3078	-0.3678	-0.2479
hsa.miR.3195	0.4110	hsa.miR.24.1	0.8768	-11.7714	5.4784e-32 ***	-0.4659	-0.5435	-0.3883

Table S2: Pairwise Comparisons of ROC Curve Areas and 0.95 Level Confidence Intervals								
Variable1	AUC1	Variable2	AUC2	Z	p.value	AUC_diff	AUC_diff_lower	AUC_diff_upper
hsa.miR.3195	0.4110	hsa.miR.548a.5p	0.5061	-2.6260	8.6390e-03 **	-0.0951	-0.1662	-0.0241
hsa.miR.3195	0.4110	hsa.miR.101	0.4732	-2.0481	4.0549e-02 *	-0.0623	-0.1219	-0.0027
hsa.miR.3195	0.4110	hsa.miR.142.3p	0.7526	-10.8225	2.6930e-27 ***	-0.3416	-0.4035	-0.2798
hsa.miR.3195	0.4110	hsa.miR.362.3p	0.8405	-13.5295	1.0477e-41 ***	-0.4296	-0.4918	-0.3673
hsa.miR.3195	0.4110	hsa.miR.29b	0.3944	0.4483	6.5396e-01	0.0166	-0.0559	0.0891
hsa.miR.3195	0.4110	hsa.miR.342.3p	0.6690	-10.5543	4.8502e-26 ***	-0.2581	-0.3060	-0.2101
hsa.miR.3195	0.4110	hsa.miR.32.5p	0.1490	5.7643	8.2002e-09 ***	0.2620	0.1729	0.3510
hsa.miR.3195	0.4110	hsa.miR.6780b.5p	0.4537	-1.6026	1.0903e-01	-0.0427	-0.0950	0.0095
hsa.miR.3195	0.4110	hsa.miR.6740.5p	0.8443	-10.7398	6.6216e-27 ***	-0.4333	-0.5124	-0.3543
hsa.miR.7641.1	0.9606	hsa.miR.494.3p	0.6782	6.5283	6.6532e-11 ***	0.2823	0.1976	0.3671
hsa.miR.7641.1	0.9606	hsa.miR.33a	0.7188	5.7588	8.4699e-09 ***	0.2418	0.1595	0.3241
hsa.miR.7641.1	0.9606	hsa.miR.24.1	0.8768	3.2735	1.0622e-03 **	0.0837	0.0336	0.1339
hsa.miR.7641.1	0.9606	hsa.miR.548a.5p	0.5061	8.8130	1.2182e-18 ***	0.4545	0.3534	0.5555
hsa.miR.7641.1	0.9606	hsa.miR.101	0.4732	10.0036	1.4695e-23 ***	0.4873	0.3918	0.5828
hsa.miR.7641.1	0.9606	hsa.miR.142.3p	0.7526	5.3895	7.0641e-08 ***	0.2080	0.1323	0.2836
hsa.miR.7641.1	0.9606	hsa.miR.362.3p	0.8405	4.1598	3.1859e-05 ***	0.1200	0.0635	0.1766

Table S2: Pairwise Comparisons of ROC Curve Areas and 0.95 Level Confidence Intervals								
Variable1	AUC1	Variable2	AUC2	Z	p.value	AUC_diff	AUC_diff_lower	AUC_diff_upper
hsa.miR.7641.1	0.9606	hsa.miR.29b	0.3944	11.3740	5.6333e-30 ***	0.5662	0.4686	0.6638
hsa.miR.7641.1	0.9606	hsa.miR.342.3p	0.6690	6.7799	1.2028e-11 ***	0.2915	0.2073	0.3758
hsa.miR.7641.1	0.9606	hsa.miR.32.5p	0.1490	23.5644	8.9428e-123 ***	0.8116	0.7441	0.8791
hsa.miR.7641.1	0.9606	hsa.miR.6780b.5p	0.4537	10.4701	1.1849e-25 ***	0.5069	0.4120	0.6018
hsa.miR.7641.1	0.9606	hsa.miR.6740.5p	0.8443	3.6401	2.7255e-04 ***	0.1163	0.0537	0.1789
hsa.miR.494.3p	0.6782	hsa.miR.33a	0.7188	-1.2427	2.1398e-01	-0.0405	-0.1045	0.0234
hsa.miR.494.3p	0.6782	hsa.miR.24.1	0.8768	-5.7951	6.8289e-09 ***	-0.1986	-0.2658	-0.1314
hsa.miR.494.3p	0.6782	hsa.miR.548a.5p	0.5061	4.4657	7.9819e-06 ***	0.1721	0.0966	0.2477
hsa.miR.494.3p	0.6782	hsa.miR.101	0.4732	7.7945	6.4674e-15 ***	0.2050	0.1535	0.2566
hsa.miR.494.3p	0.6782	hsa.miR.142.3p	0.7526	-2.6258	8.6434e-03 **	-0.0743	-0.1298	-0.0189
hsa.miR.494.3p	0.6782	hsa.miR.362.3p	0.8405	-5.3761	7.6107e-08 ***	-0.1623	-0.2214	-0.1031
hsa.miR.494.3p	0.6782	hsa.miR.29b	0.3944	8.2403	1.7181e-16 ***	0.2839	0.2164	0.3514
hsa.miR.494.3p	0.6782	hsa.miR.342.3p	0.6690	0.3771	7.0609e-01	0.0092	-0.0388	0.0572
hsa.miR.494.3p	0.6782	hsa.miR.32.5p	0.1490	11.8794	1.5138e-32 ***	0.5293	0.4419	0.6166
hsa.miR.494.3p	0.6782	hsa.miR.6780b.5p	0.4537	6.7988	1.0546e-11 ***	0.2246	0.1598	0.2893
hsa.miR.494.3p	0.6782	hsa.miR.6740.5p	0.8443	-4.0220	5.7696e-05 ***	-0.1660	-0.2470	-0.0851

Table S2: Pairwise Comparisons of ROC Curve Areas and 0.95 Level Confidence Intervals								
Variable1	AUC1	Variable2	AUC2	Z	p.value	AUC_diff	AUC_diff_lower	AUC_diff_upper
hsa.miR.33a	0.7188	hsa.miR.24.1	0.8768	-4.0821	4.4631e-05 ***	-0.1581	-0.2339	-0.0822
hsa.miR.33a	0.7188	hsa.miR.548a.5p	0.5061	4.8765	1.0796e-06 ***	0.2127	0.1272	0.2982
hsa.miR.33a	0.7188	hsa.miR.101	0.4732	7.3767	1.6222e-13 ***	0.2455	0.1803	0.3108
hsa.miR.33a	0.7188	hsa.miR.142.3p	0.7526	-0.9689	3.3260e-01	-0.0338	-0.1022	0.0346
hsa.miR.33a	0.7188	hsa.miR.362.3p	0.8405	-3.8873	1.0138e-04 ***	-0.1218	-0.1831	-0.0604
hsa.miR.33a	0.7188	hsa.miR.29b	0.3944	7.1351	9.6750e-13 ***	0.3244	0.2353	0.4135
hsa.miR.33a	0.7188	hsa.miR.342.3p	0.6690	1.6504	9.8863e-02 .	0.0498	-0.0093	0.1089
hsa.miR.33a	0.7188	hsa.miR.32.5p	0.1490	12.5313	5.0306e-36 ***	0.5698	0.4807	0.6589
hsa.miR.33a	0.7188	hsa.miR.6780b.5p	0.4537	8.1127	4.9510e-16 ***	0.2651	0.2011	0.3291
hsa.miR.33a	0.7188	hsa.miR.6740.5p	0.8443	-3.1601	1.5774e-03 **	-0.1255	-0.2034	-0.0477
hsa.miR.24.1	0.8768	hsa.miR.548a.5p	0.5061	8.4766	2.3182e-17 ***	0.3707	0.2850	0.4565
hsa.miR.24.1	0.8768	hsa.miR.101	0.4732	9.8159	9.6148e-23 ***	0.4036	0.3230	0.4842
hsa.miR.24.1	0.8768	hsa.miR.142.3p	0.7526	4.2589	2.0547e-05 ***	0.1243	0.0671	0.1814
hsa.miR.24.1	0.8768	hsa.miR.362.3p	0.8405	1.5247	1.2734e-01	0.0363	-0.0104	0.0830
hsa.miR.24.1	0.8768	hsa.miR.29b	0.3944	10.5063	8.0835e-26 ***	0.4825	0.3925	0.5725
hsa.miR.24.1	0.8768	hsa.miR.342.3p	0.6690	6.2467	4.1912e-10 ***	0.2078	0.1426	0.2730

Table S2: Pairwise Comparisons of ROC Curve Areas and 0.95 Level Confidence Intervals								
Variable1	AUC1	Variable2	AUC2	Z	p.value	AUC_diff	AUC_diff_lower	AUC_diff_upper
hsa.miR.24.1	0.8768	hsa.miR.32.5p	0.1490	20.7555	1.0942e-95 ***	0.7279	0.6591	0.7966
hsa.miR.24.1	0.8768	hsa.miR.6780b.5p	0.4537	10.2132	1.7314e-24 ***	0.4232	0.3420	0.5044
hsa.miR.24.1	0.8768	hsa.miR.6740.5p	0.8443	1.0898	2.7578e-01	0.0326	-0.0260	0.0911
hsa.miR.548a.5p	0.5061	hsa.miR.101	0.4732	0.9144	3.6049e-01	0.0329	-0.0376	0.1033
hsa.miR.548a.5p	0.5061	hsa.miR.142.3p	0.7526	-6.4175	1.3858e-10 ***	-0.2465	-0.3218	-0.1712
hsa.miR.548a.5p	0.5061	hsa.miR.362.3p	0.8405	-8.1484	3.6886e-16 ***	-0.3344	-0.4149	-0.2540
hsa.miR.548a.5p	0.5061	hsa.miR.29b	0.3944	2.5525	1.0697e-02 *	0.1117	0.0259	0.1975
hsa.miR.548a.5p	0.5061	hsa.miR.342.3p	0.6690	-4.7680	1.8610e-06 ***	-0.1629	-0.2299	-0.0959
hsa.miR.548a.5p	0.5061	hsa.miR.32.5p	0.1490	7.4756	7.6856e-14 ***	0.3571	0.2635	0.4508
hsa.miR.548a.5p	0.5061	hsa.miR.6780b.5p	0.4537	1.3654	1.7212e-01	0.0524	-0.0228	0.1277
hsa.miR.548a.5p	0.5061	hsa.miR.6740.5p	0.8443	-7.9616	1.6983e-15 ***	-0.3382	-0.4214	-0.2549
hsa.miR.101	0.4732	hsa.miR.142.3p	0.7526	-8.7988	1.3823e-18 ***	-0.2793	-0.3416	-0.2171
hsa.miR.101	0.4732	hsa.miR.362.3p	0.8405	-10.6197	2.4129e-26 ***	-0.3673	-0.4351	-0.2995
hsa.miR.101	0.4732	hsa.miR.29b	0.3944	2.0283	4.2529e-02 *	0.0789	0.0027	0.1551
hsa.miR.101	0.4732	hsa.miR.342.3p	0.6690	-7.0658	1.5974e-12 ***	-0.1958	-0.2501	-0.1415
hsa.miR.101	0.4732	hsa.miR.32.5p	0.1490	6.8680	6.5105e-12 ***	0.3243	0.2317	0.4168

Table S2: Pairwise Comparisons of ROC Curve Areas and 0.95 Level Confidence Intervals								
Variable1	AUC1	Variable2	AUC2	Z	p.value	AUC_diff	AUC_diff_lower	AUC_diff_upper
hsa.miR.101	0.4732	hsa.miR.6780b.5p	0.4537	0.6727	5.0111e-01	0.0196	-0.0374	0.0766
hsa.miR.101	0.4732	hsa.miR.6740.5p	0.8443	-8.8273	1.0724e-18 ***	-0.3710	-0.4534	-0.2887
hsa.miR.142.3p	0.7526	hsa.miR.362.3p	0.8405	-3.3399	8.3813e-04 ***	-0.0879	-0.1396	-0.0363
hsa.miR.142.3p	0.7526	hsa.miR.29b	0.3944	9.5903	8.7840e-22 ***	0.3582	0.2850	0.4314
hsa.miR.142.3p	0.7526	hsa.miR.342.3p	0.6690	3.0797	2.0723e-03 **	0.0836	0.0304	0.1368
hsa.miR.142.3p	0.7526	hsa.miR.32.5p	0.1490	16.5488	1.6328e-61 ***	0.6036	0.5321	0.6751
hsa.miR.142.3p	0.7526	hsa.miR.6780b.5p	0.4537	9.6523	4.8060e-22 ***	0.2989	0.2382	0.3596
hsa.miR.142.3p	0.7526	hsa.miR.6740.5p	0.8443	-2.5015	1.2366e-02 *	-0.0917	-0.1636	-0.0199
hsa.miR.362.3p	0.8405	hsa.miR.29b	0.3944	10.8216	2.7193e-27 ***	0.4462	0.3654	0.5270
hsa.miR.362.3p	0.8405	hsa.miR.342.3p	0.6690	7.3352	2.2133e-13 ***	0.1715	0.1257	0.2173
hsa.miR.362.3p	0.8405	hsa.miR.32.5p	0.1490	18.5306	1.1697e-76 ***	0.6915	0.6184	0.7647
hsa.miR.362.3p	0.8405	hsa.miR.6780b.5p	0.4537	11.2627	2.0057e-29 ***	0.3869	0.3195	0.4542
hsa.miR.362.3p	0.8405	hsa.miR.6740.5p	0.8443	-0.1480	8.8232e-01	-0.0038	-0.0535	0.0460
hsa.miR.29b	0.3944	hsa.miR.342.3p	0.6690	-7.9594	1.7288e-15 ***	-0.2746	-0.3423	-0.2070
hsa.miR.29b	0.3944	hsa.miR.32.5p	0.1490	5.2158	1.8298e-07 ***	0.2454	0.1532	0.3376
hsa.miR.29b	0.3944	hsa.miR.6780b.5p	0.4537	-1.5352	1.2473e-01	-0.0593	-0.1350	0.0164

Table S2: Pairwise Comparisons of ROC Curve Areas and 0.95 Level Confidence Intervals								
Variable1	AUC1	Variable2	AUC2	Z	p.value	AUC_diff	AUC_diff_lower	AUC_diff_upper
hsa.miR.29b	0.3944	hsa.miR.6740.5p	0.8443	-9.2725	1.8183e-20 ***	-0.4499	-0.5450	-0.3548
hsa.miR.342.3p	0.6690	hsa.miR.32.5p	0.1490	12.1199	8.2856e-34 ***	0.5200	0.4359	0.6041
hsa.miR.342.3p	0.6690	hsa.miR.6780b.5p	0.4537	8.0032	1.2127e-15 ***	0.2153	0.1626	0.2681
hsa.miR.342.3p	0.6690	hsa.miR.6740.5p	0.8443	-5.0979	3.4337e-07 ***	-0.1753	-0.2427	-0.1079
hsa.miR.32.5p	0.1490	hsa.miR.6780b.5p	0.4537	-6.8462	7.5838e-12 ***	-0.3047	-0.3919	-0.2175
hsa.miR.32.5p	0.1490	hsa.miR.6740.5p	0.8443	-18.2479	2.1502e-74 ***	-0.6953	-0.7700	-0.6206
hsa.miR.6780b.5p	0.4537	hsa.miR.6740.5p	0.8443	-9.3460	9.1015e-21 ***	-0.3906	-0.4725	-0.3087

Signif. codes: 0 ' *** ' 0.001 ' ** ' 0.01 ' * ' 0.05 '.' 0.1 ' ' 1

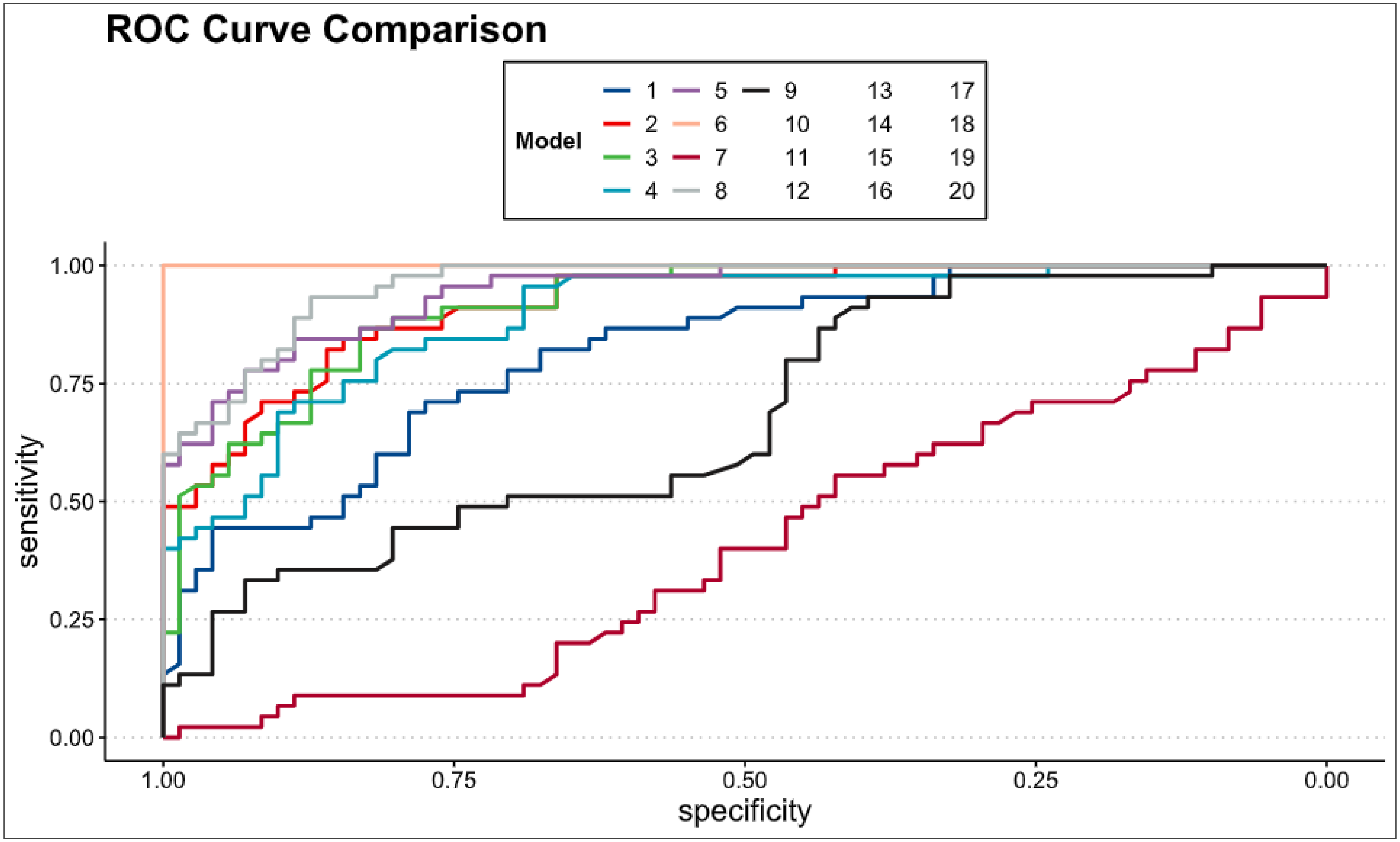

*The number of miRNA represents the number indicated in the table 1

Figure S1: Un supersvised K-means clustering of the miRNA expressiojn patterns among AMI, CAD and HC s

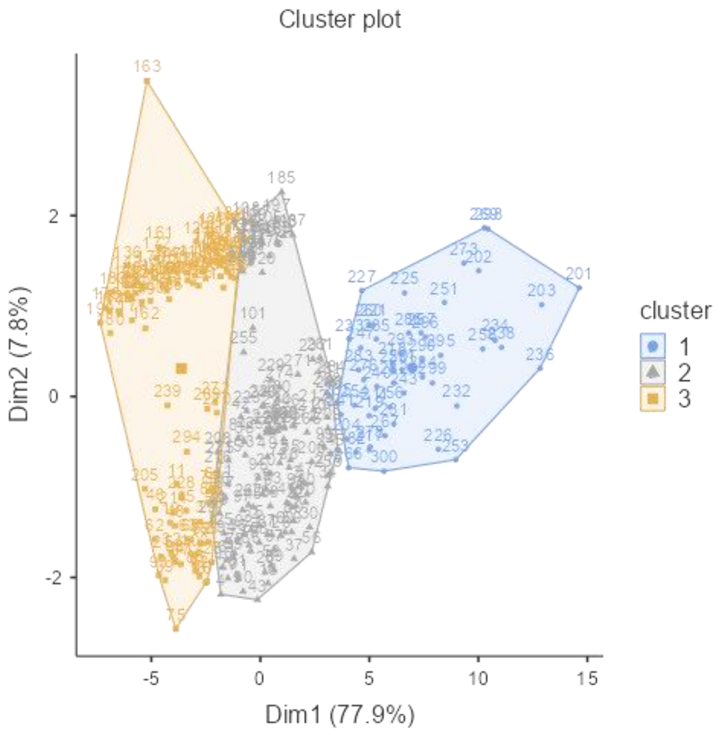

1, 2,3 Represents the cluster number formed by unsupervised machine learning.

Table S3: Contingency table for distribution of Actual group of patients among the clusters formed by machine learning

Contingency Tables

		Group of Patient			
**Clustering**		**HC**	**CAD**	**AMI**	**Total**
cluster1	Observed	**48**	**1**	**0**	**49**
	% within row	98.0 %	2.0 %	0.0 %	100.0 %
	% within column	48.0 %	1.0 %	0.0 %	16.3 %
cluster2	Observed	**12**	**38**	**67**	**117**
	% within row	10.3 %	32.5 %	57.3 %	100.0 %
	% within column	12.0 %	38.0 %	67.0 %	39.0 %
cluster3	Observed	**40**	**61**	**33**	**134**
	% within row	29.9 %	45.5 %	24.6 %	100.0 %
	% within column	40.0 %	61.0 %	33.0 %	44.7 %

Contingency Tables

Clustering		Group of Patient			
		**HC**	**CAD**	**AMI**	**Total**
Total	Observed	100	100	100	300
	% within row	33.3 %	33.3 %	33.3 %	100.0 %
	% within column	100.0 %	100.0 %	100.0 %	100.0

χ^2 Tests

	Value	df	p
χ^2	140	4	**< .001**
N	300		

HC: Healthy controls

AMI: Acute myocardial infarction

CAD: Coronary artery disease

Kruskal-Wallis Rank Sum Test comparison of miRNAs across AMI and CAD groups.

I.

Summary Statistics

TargetVariable	X.TYPE	count	min	Quantile_1st_25	mean	median	Quantile_3rd_75	max	variance	std_err	skewness	kurtosis
hsa.miR.4505	AMI	45	-1.4800	1.1400	2.3818	2.1800	3.9700	6.0900	3.9002	0.2944	-0.1014	2.3872
hsa.miR.4505	CAD	71	-4.9500	-2.0050	-0.3759	-0.4700	1.3300	4.5900	4.5151	0.2522	0.0266	2.2726

Kruskal-Wallis Test - hsa.miR.4505 By X.TYPE

Chi.squared	P.value
32.7146	1.0673e-08

Aditional Statistics For Kruskal-Wallis Test - hsa.miR.4505 By X.TYPE

Expectation	Variance	Covariance
2632.5000	31150.8900	31150.8900

II.

Summary Statistics												
**TargetVariable**	**X.TYPE**	**count**	**min**	**Quantile_1st_25**	**mean**	**median**	**Quantile_3rd_75**	**max**	**variance**	**std_err**	**skewness**	**kurtosis**
hsa.Let.7c.5p	AMI	45	-0.3800	2.6500	3.9729	3.8300	5.3100	8.5500	3.8353	0.2919	0.1608	2.6409
hsa.Let.7c.5p	CAD	71	-4.3600	-1.8800	-0.0755	0.0200	1.2750	3.9900	4.3106	0.2464	0.0539	2.0938

Kruskal-Wallis Test - hsa.Let.7c.5p By X.TYPE	
**Chi.squared**	**P.value**
57.4712	3.4306e-14

Aditional Statistics For Kruskal-Wallis Test - hsa.Let.7c.5p By X.TYPE		
**Expectation**	**Variance**	**Covariance**
2632.5000	31150.2900	31150.2900

III.

Summary Statistics												
**TargetVariable**	**X.TYPE**	**count**	**min**	**Quantile_1st_25**	**mean**	**median**	**Quantile_3rd_75**	**max**	**variance**	**std_err**	**skewness**	**kurtosis**
hsa.miR.6875.5p	AMI	45	0.4800	2.6900	3.9031	3.9000	4.8900	7.7700	3.0610	0.2608	0.2115	2.4768
hsa.miR.6875.5p	CAD	71	-4.6800	-1.7450	-0.0344	0.1700	1.7250	5.0200	5.2703	0.2724	-0.1451	2.1937

Kruskal-Wallis Test - hsa.miR.6875.5p By X.TYPE	
**Chi.squared**	**P.value**
56.6142	5.3069e-14

Aditional Statistics For Kruskal-Wallis Test - hsa.miR.6875.5p By X.TYPE		
**Expectation**	**Variance**	**Covariance**
2632.5000	31150.8900	31150.8900

IV.

Summary Statistics

TargetVariable	X.TYPE	count	min	Quantile_1st_25	mean	median	Quantile_3rd_75	max	variance	std_err	skewness	kurtosis
hsa.miR.939	AMI	45	-1.9700	2.1500	3.7287	3.4200	5.2900	7.9400	4.6820	0.3226	0.0328	2.6721
hsa.miR.939	CAD	71	-4.5400	-1.8350	-0.0331	-0.1100	1.6500	4.7300	4.7691	0.2592	0.0978	2.1980

Kruskal-Wallis Test - hsa.miR.939 By X.TYPE	
**Chi.squared**	**P.value**
50.5211	1.1789e-12

Aditional Statistics For Kruskal-Wallis Test - hsa.miR.939 By X.TYPE		
**Expectation**	**Variance**	**Covariance**
2632.5000	31150.7700	31150.7700

V.

Summary Statistics

TargetVariable	X.TYPE	count	min	Quantile_1st_25	mean	median	Quantile_3rd_75	max	variance	std_err	skewness	kurtosis
hsa.let.7b	AMI	45	0.0600	2.7500	4.4731	4.3500	6.1500	8.4000	4.1210	0.3026	0.0005	2.0484
hsa.let.7b	CAD	71	-4.2600	-2.0250	-0.2065	0.0000	1.4600	3.9100	4.8165	0.2605	-0.1284	2.0812

Kruskal-Wallis Test - hsa.let.7b By X.TYPE	
**Chi.squared**	**P.value**
64.5021	9.9920e-16

Aditional Statistics For Kruskal-Wallis Test - hsa.let.7b By X.TYPE		
**Expectation**	**Variance**	**Covariance**
2632.5000	31151.0100	31151.0100

Vi.

Summary Statistics

TargetVariable	X.TYPE	count	min	Quantile_1st_25	mean	median	Quantile_3rd_75	max	variance	std_err	skewness	kurtosis
hsa.miR.4485.3p	AMI	45	3.9200	5.9700	7.8449	7.6900	9.5000	11.8100	4.1548	0.3039	0.0871	2.0425
hsa.miR.4485.3p	CAD	71	-4.1700	-1.1550	-0.0520	0.1300	1.3400	3.6000	3.9033	0.2345	-0.2317	2.2351

Kruskal-Wallis Test - hsa.miR.4485.3p By X.TYPE	
**Chi.squared**	**P.value**
81.9250	0

Aditional Statistics For Kruskal-Wallis Test - hsa.miR.4485.3p By X.TYPE		
**Expectation**	**Variance**	**Covariance**
2632.5000	31150.5300	31150.5300

Vii.

Summary Statistics												
**TargetVariable**	**X.TYPE**	**count**	**min**	**Quantile_1st_25**	**mean**	**median**	**Quantile_3rd_75**	**max**	**variance**	**std_err**	**skewness**	**kurtosis**

Summary Statistics												
**TargetVariable**	**X.TYPE**	**count**	**min**	**Quantile_1st_25**	**mean**	**median**	**Quantile_3rd_75**	**max**	**variance**	**std_err**	**skewness**	**kurtosis**
hsa.miR.3195	AMI	45	-0.6900	1.2900	2.9611	3.3700	4.5100	6.9800	3.7856	0.2900	-0.0906	2.1495
hsa.miR.3195	CAD	71	-0.2800	1.6500	3.5445	3.6800	5.0600	7.3100	4.0229	0.2380	-0.1495	1.8605

Kruskal-Wallis Test - hsa.miR.3195 By X.TYPE	
**Chi.squared**	**P.value**
2.5984	0.1070

Aditional Statistics For Kruskal-Wallis Test - hsa.miR.3195 By X.TYPE		
**Expectation**	**Variance**	**Covariance**
2632.5000	31150.2900	31150.2900

Viii.

Summary Statistics

TargetVariable	X.TYPE	count	min	Quantile_1st_25	mean	median	Quantile_3rd_75	max	variance	std_err	skewness	kurtosis
hsa.miR.7641.1	AMI	45	1.6000	3.0500	4.8484	5.5100	6.3600	8.4100	3.6087	0.2832	-0.1504	1.8022
hsa.miR.7641.1	CAD	71	-4.3400	-1.4100	-0.1862	-0.0100	1.5550	4.6100	4.6680	0.2564	-0.0698	2.3529

Kruskal-Wallis Test - hsa.miR.7641.1 By X.TYPE	
**Chi.squared**	**P.value**
69.5120	1.1102e-16

Aditional Statistics For Kruskal-Wallis Test - hsa.miR.7641.1 By X.TYPE		
**Expectation**	**Variance**	**Covariance**
2632.5000	31150.1700	31150.1700

iX.

Summary Statistics												
**TargetVariable**	**X.TYPE**	**count**	**min**	**Quantile_1st_25**	**mean**	**median**	**Quantile_3rd_75**	**max**	**variance**	**std_err**	**skewness**	**kurtosis**

Summary Statistics												
**TargetVariable**	**X.TYPE**	**count**	**min**	**Quantile_1st_25**	**mean**	**median**	**Quantile_3rd_75**	**max**	**variance**	**std_err**	**skewness**	**kurtosis**
hsa.miR.494.3p	AMI	45	0.5400	3.3200	4.6216	4.5100	6.4700	8.0400	3.3452	0.2727	-0.0127	1.9704
hsa.miR.494.3p	CAD	71	-0.2900	1.3400	3.2635	3.8000	4.7700	6.8100	4.0653	0.2393	-0.0408	1.7914

Kruskal-Wallis Test - hsa.miR.494.3p By X.TYPE	
**Chi.squared**	**P.value**
10.4121	0.0013

Aditional Statistics For Kruskal-Wallis Test - hsa.miR.494.3p By X.TYPE		
**Expectation**	**Variance**	**Covariance**
2632.5000	31149.4500	31149.4500

X.

Summary Statistics

TargetVariable	X.TYPE	count	min	Quantile_1st_25	mean	median	Quantile_3rd_75	max	variance	std_err	skewness	kurtosis
hsa.miR.33a	AMI	45	-0.3800	3.4400	5.0558	5.0300	7.0000	10.7400	5.5042	0.3497	-0.0881	2.6202
hsa.miR.33a	CAD	71	-0.9300	1.3400	3.2051	3.4900	4.7400	7.0800	4.3479	0.2475	-0.1228	1.9619

Kruskal-Wallis Test - hsa.miR.33a By X.TYPE	
**Chi.squared**	**P.value**
15.6853	7.4802e-05

Aditional Statistics For Kruskal-Wallis Test - hsa.miR.33a By X.TYPE		
**Expectation**	**Variance**	**Covariance**
2632.5000	31150.1700	31150.1700

Xi.

Summary Statistics

TargetVariable	X.TYPE	count	min	Quantile_1st_25	mean	median	Quantile_3rd_75	max	variance	std_err	skewness	kurtosis
hsa.miR.24.1	AMI	45	2.8500	5.0500	6.7456	6.4000	8.7000	11.1900	4.7945	0.3264	0.3408	2.0379
hsa.miR.24.1	CAD	71	-0.0500	1.4650	3.2293	3.7500	5.0150	6.6300	3.7505	0.2298	-0.1300	1.7737

Kruskal-Wallis Test - hsa.miR.24.1 By X.TYPE	
**Chi.squared**	**P.value**
46.5358	8.9958e-12

Aditional Statistics For Kruskal-Wallis Test - hsa.miR.24.1 By X.TYPE		
**Expectation**	**Variance**	**Covariance**
2632.5000	31150.5300	31150.5300

Xii.

Summary Statistics												
**TargetVariable**	**X.TYPE**	**count**	**min**	**Quantile_1st_25**	**mean**	**median**	**Quantile_3rd_75**	**max**	**variance**	**std_err**	**skewness**	**kurtosis**

Summary Statistics												
**TargetVariable**	**X.TYPE**	**count**	**min**	**Quantile_1st_25**	**mean**	**median**	**Quantile_3rd_75**	**max**	**variance**	**std_err**	**skewness**	**kurtosis**
hsa.miR.548a.5p	AMI	45	-1.1000	1.7800	3.4744	2.9700	5.0400	9.5000	6.9427	0.3928	0.6329	2.9527
hsa.miR.548a.5p	CAD	71	-0.5300	1.3350	3.1997	3.7200	4.6500	6.8200	3.9101	0.2347	-0.1769	1.8950

Kruskal-Wallis Test - hsa.miR.548a.5p By X.TYPE	
**Chi.squared**	**P.value**
0.0122	0.9120

Aditional Statistics For Kruskal-Wallis Test - hsa.miR.548a.5p By X.TYPE		
**Expectation**	**Variance**	**Covariance**
2632.5000	31150.6500	31150.6500

Xiii.

Summary Statistics

TargetVariable	X.TYPE	count	min	Quantile_1st_25	mean	median	Quantile_3rd_75	max	variance	std_err	skewness	kurtosis
hsa.miR.101	AMI	45	-1.1400	1.9800	3.1193	2.7900	4.7300	7.5800	4.3595	0.3113	-0.0225	2.5185
hsa.miR.101	CAD	71	-0.4000	1.4800	3.3442	3.8200	4.9150	7.0800	3.9661	0.2363	-0.1237	1.8294

Kruskal-Wallis Test - hsa.miR.101 By X.TYPE	
**Chi.squared**	**P.value**
0.2347	0.6281

Aditional Statistics For Kruskal-Wallis Test - hsa.miR.101 By X.TYPE		
**Expectation**	**Variance**	**Covariance**
2632.5000	31150.0500	31150.0500

Xiv.

Summary Statistics

TargetVariable	X.TYPE	count	min	Quantile_1st_25	mean	median	Quantile_3rd_75	max	variance	std_err	skewness	kurtosis
hsa.miR.142.3p	AMI	45	2.0200	3.7500	5.2724	4.8200	6.8400	9.6000	3.6739	0.2857	0.2951	2.1575
hsa.miR.142.3p	CAD	71	-0.7300	1.4750	3.1993	3.4600	4.8350	6.7100	4.0766	0.2396	-0.1511	1.8924

Kruskal-Wallis Test - hsa.miR.142.3p By X.TYPE	
**Chi.squared**	**P.value**
20.9067	4.8221e-06

Aditional Statistics For Kruskal-Wallis Test - hsa.miR.142.3p By X.TYPE		
**Expectation**	**Variance**	**Covariance**
2632.5000	31150.2900	31150.2900

Xv.

Summary Statistics

TargetVariable	X.TYPE	count	min	Quantile_1st_25	mean	median	Quantile_3rd_75	max	variance	std_err	skewness	kurtosis
hsa.miR.362.3p	AMI	45	1.6400	4.7800	6.1549	6.3400	7.6500	9.7300	3.6564	0.2851	-0.2264	2.4090
hsa.miR.362.3p	CAD	71	-0.7300	1.5100	3.2517	3.5500	4.8700	6.9200	4.1608	0.2421	0.0131	1.8492

Kruskal-Wallis Test - hsa.miR.362.3p By X.TYPE	
**Chi.squared**	**P.value**
38.0011	7.0706e-10

Aditional Statistics For Kruskal-Wallis Test - hsa.miR.362.3p By X.TYPE		
**Expectation**	**Variance**	**Covariance**
2632.5000	31150.2900	31150.2900

Xvi.

Summary Statistics												
**TargetVariable**	**X.TYPE**	**count**	**min**	**Quantile_1st_25**	**mean**	**median**	**Quantile_3rd_75**	**max**	**variance**	**std_err**	**skewness**	**kurtosis**

Summary Statistics												
**TargetVariable**	**X.TYPE**	**count**	**min**	**Quantile_1st_25**	**mean**	**median**	**Quantile_3rd_75**	**max**	**variance**	**std_err**	**skewness**	**kurtosis**
hsa.miR.29b	AMI	45	-2.4400	1.1800	2.4436	2.3100	3.7600	7.4000	4.6322	0.3208	0.2585	2.7646
hsa.miR.29b	CAD	71	-0.4100	1.4700	3.2185	3.3900	4.9000	6.9900	3.9331	0.2354	-0.1284	1.8881

Kruskal-Wallis Test - hsa.miR.29b By X.TYPE	
**Chi.squared**	**P.value**
3.6567	0.0558

Aditional Statistics For Kruskal-Wallis Test - hsa.miR.29b By X.TYPE		
**Expectation**	**Variance**	**Covariance**
2632.5000	31150.1700	31150.1700

Xvii.

Summary Statistics

TargetVariable	X.TYPE	count	min	Quantile_1st_25	mean	median	Quantile_3rd_75	max	variance	std_err	skewness	kurtosis
hsa.miR.342.3p	AMI	45	0.7100	3.1400	4.4760	4.5100	5.7500	7.4500	3.3879	0.2744	-0.2426	2.2906
hsa.miR.342.3p	CAD	71	-0.2000	1.7450	3.2649	3.4600	4.7050	6.8500	3.9031	0.2345	-0.0995	1.9742

Kruskal-Wallis Test - hsa.miR.342.3p By X.TYPE	
**Chi.squared**	**P.value**
9.3611	0.0022

Aditional Statistics For Kruskal-Wallis Test - hsa.miR.342.3p By X.TYPE		
**Expectation**	**Variance**	**Covariance**
2632.5000	31150.2900	31150.2900

Xviii.

Summary Statistics												
**TargetVariable**	**X.TYPE**	**count**	**min**	**Quantile_1st_25**	**mean**	**median**	**Quantile_3rd_75**	**max**	**variance**	**std_err**	**skewness**	**kurtosis**

Summary Statistics												
**TargetVariable**	**X.TYPE**	**count**	**min**	**Quantile_1st_25**	**mean**	**median**	**Quantile_3rd_75**	**max**	**variance**	**std_err**	**skewness**	**kurtosis**
hsa.miR.32.5p	AMI	45	-8.6100	-6.5000	-4.4224	-4.2700	-2.2100	0.3100	6.4904	0.3798	0.1573	1.9623
hsa.miR.32.5p	CAD	71	-4.4900	-2.6700	-0.9004	-0.6400	0.7500	2.8700	3.9043	0.2345	-0.0243	1.7731

Kruskal-Wallis Test - hsa.miR.32.5p By X.TYPE	
**Chi.squared**	**P.value**
40.3766	2.0943e-10

Aditional Statistics For Kruskal-Wallis Test - hsa.miR.32.5p By X.TYPE		
**Expectation**	**Variance**	**Covariance**
2632.5000	31150.7700	31150.7700

XiX,

Summary Statistics

TargetVariable	X.TYPE	count	min	Quantile_1st_25	mean	median	Quantile_3rd_75	max	variance	std_err	skewness	kurtosis
hsa.miR.6780b.5p	AMI	45	-1.2000	1.1800	2.9504	3.2300	4.2900	7.2000	4.1460	0.3035	0.0135	2.3229
hsa.miR.6780b.5p	CAD	71	-0.4500	1.3650	3.2385	3.7700	4.8150	6.6400	3.8704	0.2335	-0.1724	1.8634

Kruskal-Wallis Test - hsa.miR.6780b.5p By X.TYPE	
**Chi.squared**	**P.value**
0.7032	0.4017

Aditional Statistics For Kruskal-Wallis Test - hsa.miR.6780b.5p By X.TYPE		
**Expectation**	**Variance**	**Covariance**
2632.5000	31149.5700	31149.5700

XX

Summary Statistics												
**TargetVariable**	**X.TYPE**	**count**	**min**	**Quantile_1st_25**	**mean**	**median**	**Quantile_3rd_75**	**max**	**variance**	**std_err**	**skewness**	**kurtosis**
hsa.miR.6740.5p	AMI	45	2.0300	4.5400	5.9429	6.0600	7.5100	11.0400	4.7451	0.3247	0.0962	2.5594
hsa.miR.6740.5p	CAD	71	-1.0100	1.4400	2.8994	3.0700	4.4600	6.6700	4.0159	0.2378	0.0324	2.0992

Kruskal-Wallis Test - hsa.miR.6740.5p By X.TYPE	
**Chi.squared**	**P.value**
38.8442	4.5901e-10

Aditional Statistics For Kruskal-Wallis Test - hsa.miR.6740.5p By X.TYPE		
**Expectation**	**Variance**	**Covariance**
2632.5000	31150.0500	31150.0500
